# Annual 30-m maps of global grassland class and extent (2000–2022) based on spatiotemporal Machine Learning

**DOI:** 10.1038/s41597-024-04139-6

**Published:** 2024-12-11

**Authors:** Leandro Parente, Lindsey Sloat, Vinicius Mesquita, Davide Consoli, Radost Stanimirova, Tomislav Hengl, Carmelo Bonannella, Nathália Teles, Ichsani Wheeler, Maria Hunter, Steffen Ehrmann, Laerte Ferreira, Ana Paula Mattos, Bernard Oliveira, Carsten Meyer, Murat Şahin, Martijn Witjes, Steffen Fritz, Ziga Malek, Fred Stolle

**Affiliations:** 1 OpenGeoHub Foundation, Doorwerth, The Netherlands; 2 Land & Carbon Lab, World Resources Institute, Washington, DC USA; 3 Remote Sensing and GIS Laboratory (LAPIG/UFG), Goiânia, Brazil; 4 Laboratory of Geo-Information Science and Remote Sensing, Wageningen University & Research, Wageningen, The Netherlands; 5 International Institute for Applied Systems Analysis (IIASA), Laxenburg, Austria; 6 German Centre for Integrative Biodiversity Research (iDiv) Halle-Jena-Leipzig, Leipzig, Germany; 7 Institute of Biology, Leipzig University, Leipzig, Germany; 8 Institute of Geosciences and Geography, Martin Luther University Halle-Wittenberg, Halle, Saale Germany

**Keywords:** Environmental impact, Research data

## Abstract

The paper describes the production and evaluation of global grassland extent mapped annually for 2000–2022 at 30 m spatial resolution. The dataset showing the spatiotemporal distribution of cultivated and natural/semi-natural grassland classes was produced by using GLAD Landsat ARD-2 image archive, accompanied by climatic, landform and proximity covariates, spatiotemporal machine learning (per-class Random Forest) and over 2.3 M reference samples (visually interpreted in Very High Resolution imagery). Custom probability thresholds (based on five-fold spatial cross-validation) were used to derive dominant class maps with balanced user’s and producer’s accuracy, resulting in f1 score of 0.64 and 0.75 for cultivated and natural/semi-natural grassland, respectively. The produced maps (about 4 TB in size) are available under an open data license as Cloud-Optimized GeoTIFFs and as Google Earth Engine assets. The suggested uses of data include (1) integration with other compatible land cover products and (2) tracking the intensity and drivers of conversion of land to cultivated grasslands and from natural / semi-natural grasslands into other land use systems.

## Background & Summary

Grasslands are among the most vital global ecosystems, and, comprising open grasslands, grassy shrublands, and savannas, they cover approximately 40% of the Earth’s surface^1,2^. These ecosystems are critical for carbon sequestration, food production, biodiversity maintenance, and cultural heritage for people all over the world^1^. Klein *et al*.^3^ estimate that in 2000, there were 3,322 Mha of pastures in the world, both pastures and croplands experiencing rapid expansion. However, despite their ecological, cultural and socioeconomic importance, no comprehensive time series of high-resolution global maps specifically focused on grasslands yet exists. In addition, more detailed information on grassland management and use is also lacking, particularly at high resolutions and over extended periods of time. Geospatial monitoring for these areas is urgently needed to support conservation efforts, to underpin meaningful corporate supply chain no-conversion commitments, to reduce greenhouse gas emissions from the land sector^4,5^, to aid contribution to positive land use planning, allow finance for nature-based solutions and to contribute to restoring degraded landscapes^1,2^.

Grasslands are one of the most challenging classes in land cover monitoring, driven by various natural, anthropogenic, and social aspects that vary between regions and cultures^6^. General-purpose global land cover maps have traditionally mapped classes such as grasslands and shrublands with coarse spatial resolution, such as 500 m for NASA’s Global Land Cover Type^7^ and 300 m for ESA’s Climate Change Initiative Land Cover^8^. Other products such as HYDE (10 km)^3^, Earthstat (10 km)^9^, and HILDA+ (1 km)^10^ further differentiate grassland management systems such as pastures/rangelands and unmanaged lands. However, their spatial resolution remains relatively coarse. In addition, the loose class definitions of existing grassland maps significantly hinder interoperability between classification systems. Recently, higher-resolution general-purpose land cover maps have become available by classifying Landsat (30 m) and Sentinel-2 (10 m) Earth Observation (EO) archives^11–15^, improving spatial resolution of grasslands, however have maintained the broad definition for grasslands without incorporating information on how they are actually intended to be used; thus limiting their usability for farmers, national agencies monitoring livestock, and agricultural extension experts. National medium- to high-resolution products^16,17^ successfully add further differentiation to grasslands, but unfortunately cannot be used globally due to their limited spatial coverage.

In response to the need for detailed global-scale monitoring products targeting grasslands, the Land & Carbon Lab initiated the Global Pasture Watch (GPW) research consortium, gathering experts from the World Resources Institute (WRI), OpenGeoHub Foundation, the Image Processing and GIS Laboratory at the Federal University of Goiás (LAPIG/UFG), the International Institute for Applied Systems Analysis (IIASA), the German Center for Integrative Biodiversity Research (iDiv), Cornell University; and the Global Land Analysis and Discovery laboratory of the University of Maryland (GLAD). GPW aims to advance grassland monitoring by creating recurrent collections of global mapping products from the year 2000 onward at a suitable spatial resolution (*i.e*. 30 m) to create fit-for-purpose monitoring solutions which are uniquely designed to be open to incorporating the significantly regional cultural knowledge surrounding grasslands.

In this paper, we present a novel data set with annual time series of global cultivated and natural/semi-natural grasslands mapped at 30 m spatial resolution covering the period from 2000 to 2022. We first explain all sampling and modeling steps and then report results of spatial cross-validation and comparison with existing datasets (*e.g*. GLanCE^18^, UMD GLAD GLCLUC^13^, GLC_FCS30D^15^). We also visualize the annual values of the dominant class and the probability of grasslands, discuss potential applications, and openly report the limitations and future needs of the data we have produced. The data are available under open license (CC-BY) and will be regularly updated and improved with additional regional contexts, as well as new years added as the EO images become available.

## Methods

**Fig. 1:**
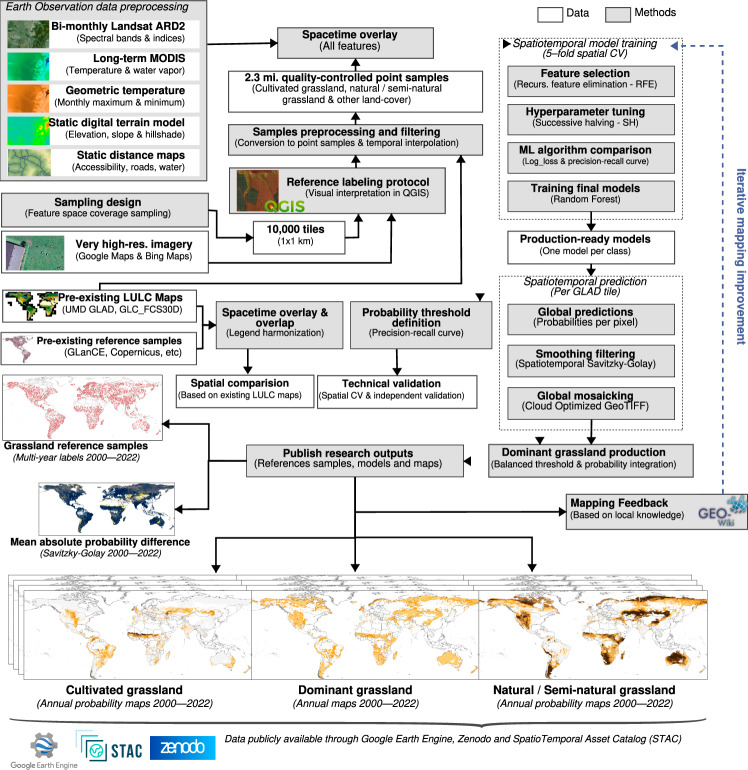
The Global Pasture Watch grassland mapping framework encompasses general processing workflows, key inputs and outputs, and a feedback loop to improve future versions of the global maps.

Our mapping framework, shown in Fig. 1, was based on multiple Earth Observation (EO) data such as GLAD Landsat ARD-2^19^, MOD11A2^20^, MCD19A2^21^, digital terrain model derivatives and distance maps of accessibility, roads, and water. To train the models, we used more than 2.3 M reference samples visually interpreted in Very High Resolution (VHR) images (*i.e*. Google Maps and Bing Maps). Two independent spatiotemporal machine learning (ML) models^22^ were used to predict each grassland class (*i.e. cultivated grassland* and *natural/semi-natural grassland*) over multiple years on a global scale. We produced predictions for all years from 2000 to 2022, resulting in a time series of global probability maps for cultivated and natural/semi-natural grassland at 30 m spatial resolution. Both probabilities were used to derive an integrated dominant class of grasslands, considering a custom global threshold per class. The exact methodological steps are described in the following sections.

### Reference sampling design

We use a Feature Space Coverage Sampling (FSCS^23^) to generate reference samples. This sampling design helps improve the representativeness of reference samples and is especially suitable for fitting multivariate predictive mapping models^23^. We used FSCS to generate 10,000 sample tiles (*i.e*. 1 × 1 km) distributed across the World. We used 87 input layers for FSCS, shown in Table 1, restricted by a short vegetation mask that includes all pixels mapped as mosaic, shrubland, grassland, and sparse vegetation in at least one year from 1993 to 2021 (*i.e*. 13 land cover classes described in Table S1), according to the ESA/CCI global land cover time-series^24^.

**Table 1:** Input layers for the Feature Space Coverage Sampling (FSCS).

Theme	Product	Variable	Time period	Number of layers
Terrain	GLO-90 Copernicus Digital Elevation Model^84^	Elevation	2011 and 2015	1
Terrain	Geomorpho90m^85^	Slope	2018	1
Vegetation index	MODIS MOD13Q1 v061^86^	Long-term median EVI (all months)	2000 to 2021	12
		Long-term std. deviation EVI (all months)		12
Land Temperature	MODIS MOD11A2 v061^20^	Long-term median day time LST (all months)	2000 to 2021	12
		Long-term std. day time LST (all months)		12
		Long-term median night-time LST (all months)		12
		Long-term std. night time LST (all months)		12
Climate	CHELSA time-series^87^	Long-term mean precipitation (all months)	1981 to 2018	12
Water	JRC Global Surface Water^88^	Water occurrence	1984 to 2018	1
Total number of layers				87

All layers were resampled to 1 km by average and filtered by a short vegetation mask based on ESA/CCI global land cover maps^24^. The long-term derivatives were calculated considering the entire time period and a specific month (*e.g*. all Januaries from 2000 to 2021).

In practice, the FSCS steps^25^ include:

1. Principal Components Analysis (PCA) using all input layers,
2. Selection of the 10 first components (explaining 75% of variance),
3. K-Means with 10,000 clusters (targeted number of samples),
4. Calculation of Euclidean distance (in the principal component space) of all 1 km pixels to the centre of each cluster,
5. Selection of the pixel with the shortest distance for each cluster,
6. Conversion of the selected pixels to sample tiles (1 × 1 km).

### Reference labeling protocol

**Fig. 2:**
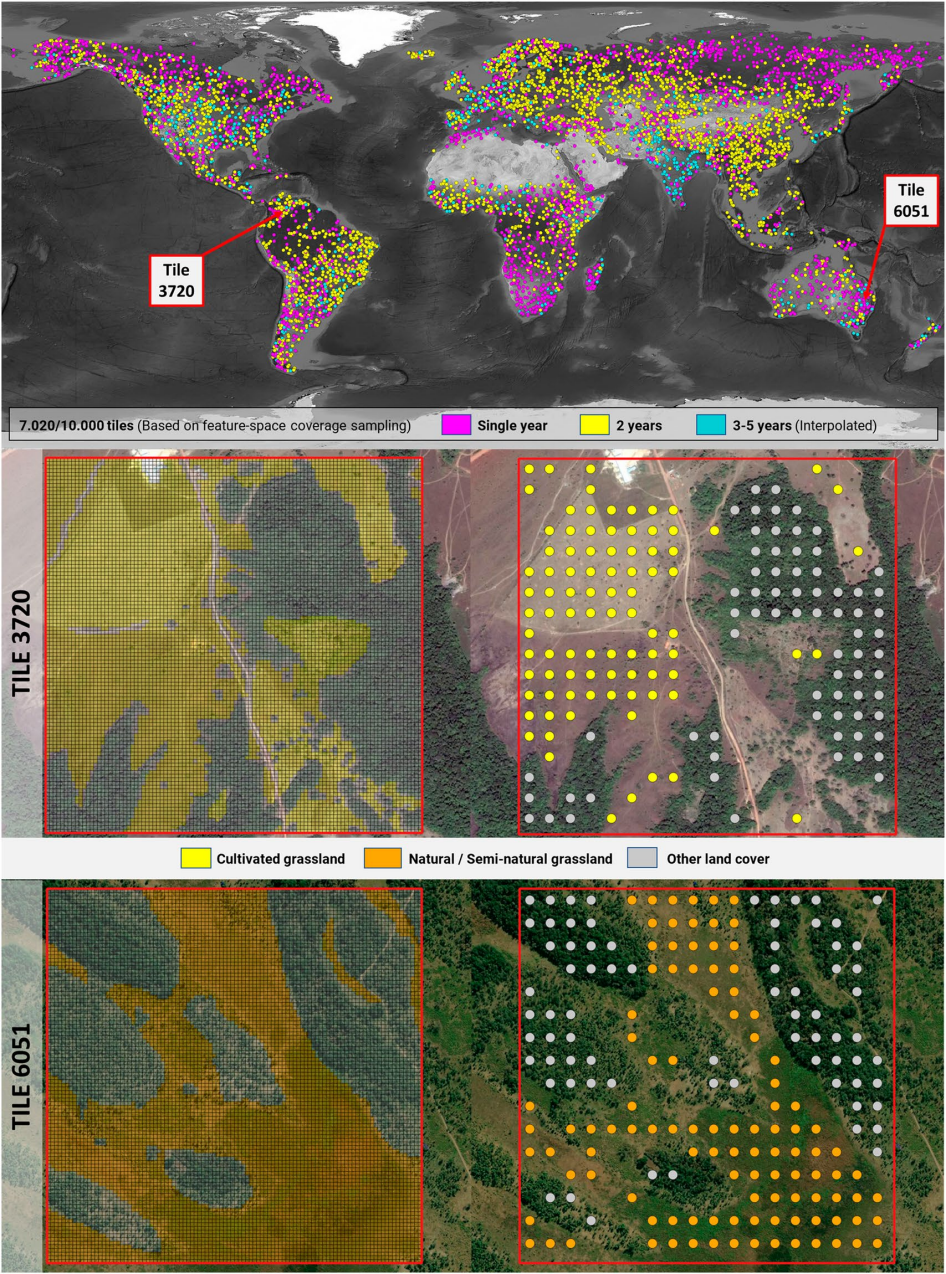
Spatial distribution of tiles with available information (Single, 2 or more years) and examples of raw interpreted and converted to points for training the prediction models.

The selected FSCS tiles were visually interpreted by 16 visual interpretation (VI) analysts who classified the entire tile surface into three classes (*i.e. cultivated grassland*, *natural/semi-natural grassland* and *other land cover*) using Google Maps and Bing Maps imagery as reference. The analysts used a QGIS plugin (https://plugins.qgis.org/plugins/qgis-fgi-plugin) specifically designed to optimize the classification process and evaluated 10,000 tile samples (*i.e*. 1 × 1 km). For each tile, the plugin automatically created a finer grid (*i.e*. 10 m grid cells), where each analyst manually assigned a single class and a reference date for a group of grid cells according to base imagery, as shown in Fig. 2. For Google Maps images, the analysts got the reference date from Google Earth software, and for Bing Maps, the plugin retrieved it through the Bing API. A total of 2,995 tiles were discarded due to a lack of suitable VHR images, predominately occurring in regions with latitudes higher than 60.5 degrees north.

### Reference labeling criteria

In order to initially capture the inherent complexity of grasslands ecosystems, we developed a hierarchical ontology based on^26^ (see Table S2) and in line with attempting to separate natural/semi-natural grasslands without significant human directed management, from those under heavy management and/or entirely cultivated grasslands. We defined grassland as any land cover type which contains at least 30% of dry or wet low vegetation, dominated by grasses and forbs (less than 3 meters) and a:

- maximum of 50% tree canopy cover (greater than 5 meters),
- maximum of 70% of other woody vegetation (scrubs and open shrubland), and
- maximum of 50% active cropland cover in mosaic landscapes of cropland and other vegetation.
  The reference labelling criteria were by necessity focused only on two end-member states (*i.e. cultivated* and *natural/semi-natural*) taking into consideration features that can be objectively identified in VHR imagery (see Fig. S1). The reference labelling criteria, shown in Table 2, was used to train all analysts to visually distinguish our mapping classes according to the follow descriptions:

**Table 2:** Visual interpretation criteria used in the reference labeling protocol.

Criteria	Cultivated grasslands	Semi-natural/natural grasslands
**Colour & texture variation**		
Short range variation	Colour/texture are geometrically regularised, high homogeneity indicative of species and/or temporal management of vegetation.	Colour/texture variations are pronounced, naturalistic patterning indicating a diversity of vegetation responding to soil/water variations.
Long range variation	Landscapes are unnaturally uniform due to management activities, disregarding soil/water variations.	Landscapes & reflect soil/water variations, are ordered with natural patterning &. plant variation
Seasonal variation	High between field heterogeneity within & between seasons.	Seasonal progression visible for similar looking vegetation types.
**Human influence & management**		
Animals	Presence of domesticated animals.	Domesticated animals mostly absent.
Animal infrastructure	Structures, enclosures, access roads indicate active management.	Human management structures are mostly absent.
Short range management	Clear geographically zoned schedules for plowing, mowing.	Absence of imposed management infrastructure at the field scale.
Long range management	Infrastructure to serve multiple fields / properties (*e.g*. access roads, watering lines).	Visually connected to natural landscape with little evidence of imposed management.
Temporal management	Long mixed farming rotations, typically managed over 2-5 years.	Continued grassland presence when inspecting several seasons.
**Contextual analysis**		
Proximity	Co-location with cropping lands likely indicates intensive management.	Distance from human accessibility indicates more naturalness.

Short-range variation refers to distances of 10 s to 100 s of meters, while long-range variation covers areas beyond 1 km, encompassing a 9 km^2^ landscape context.
- **Cultivated grassland** includes areas where grasses and other forage plants have been intentionally planted and managed, as well as areas of native grassland-type vegetation where they clearly exhibit active and ‘heavy’ management for specific human-directed uses, such as directed grazing of livestock. Many natural/semi-natural landscapes exist on a human intervention gradient, which is assumed by our criteria to initially be indicated by the presence of livestock-related infrastructure such as fencing and watering points. As interventions become more intensive through time, practices such as regular seeding, ploughing, mowing, fertilization, controlled grazing, and sometimes irrigation, aimed at enhancing productivity and maintaining the desired vegetation cover, start to become visible and/or implied by the visual character of the landscape. In general, the nonexclusive criteria applied to this class can be approximated from Table 2,
- **Natural/semi-natural grassland** includes relatively undisturbed native grasslands/short-height vegetation, such as steppes and tundra, as well as areas that have experienced varying degrees of human activity in the past. These grasslands may contain a mix of native and introduced species due to historical land use and natural processes. In general, they exhibit natural-looking patterns of varied vegetation and clearly ordered hydrological relationships throughout the landscape. This class also includes land that may have become degraded due to overuse or mismanagement but is not currently under intensive restoration or active management. Semi-natural areas may still have minimal active management and low-intensity practices such as periodic burning or episodic grazing under human direction to maintain the current grassy state or as part of arid or semi-arid transhumance practices. In general, the nonexclusive criteria applied to this class can be approximated from Table 2,
- **Other land cover** includes all other classes of land cover and land use, including, but not limited to, water bodies, rivers, snow, permanent ice, built-up areas, forest, annual crops (*e.g*. soybean, maize), perennial crops (*e.g*. coffee), bare ground, rocky outcrops, and wetlands. The definitions of the criteria may vary according to the types of LULC classes. Generally, we considered everything that does not fit into the other two classes as *Other land cover*.

Our reference labelling criteria were re-evaluated and refined through iterative discussions involving the GPW team, and may be actively fed by external analysts/users bringing additional cultural and regional expert knowledge, systematically contributing for improvements in our grassland reference samples.

### Reference sample pre-processing and filtering

All classified tiles with an assigned reference date were converted to point samples considering a 60 m of spatial support (*i.e*. two Landsat pixels). For each point sample, we derive a class proportion based on the number of grid cells (*i.e*. 10 m) for each class. For example, a point sample with 30 grid cells classified as *cultivated grassland* had a class proportion equal to 0.83 (*i.e*. 30 divided by 36). Since we implemented an independent binary classification model per grassland class, we kept only point samples with the 100% class proportion in our reference set, aiming for predictions based on distinct classes.

For point samples visually interpreted in two years (*i.e*. different reference dates for Bing Maps and Google Maps), we implemented a data augmentation approach to increase the number of samples in consecutive years in our model. Every point sample with the same class according to Bing Maps and Google Maps, and less than 5 years of time difference, was replicated in all intermediate years. For example, a point sample of *cultivated grassland* in 2010, according to Google Maps, and in 2014, according to Bing Maps, was replicated in 2011, 2012 and 2013. Assuming a minimum rotation period of 5 years for crops and grasslands^27^, this approach resulted in approximately 300,000 additional samples, mostly located in Europe, the U.S., India and South America.

The point samples were filtered considering the disagreement between our reference classes and three global land cover products (*i.e*. UMD GLAD GLCLUC^13^, GLC_FCS30D^15^ and ESA WorldCover 2020^14^), from which we obtained the mapped classes for multiple years (*i.e*. 2000, 2005, 2010, 2015 and 2020). All samples of *cultivated grassland* and *natural/semi-natural grassland* mapped as urban areas, forest, cropland, water, snow, or wetlands were removed by at least two global products in two years. Likewise, all samples of *other land cover* predicted as grassland, short vegetation or herbaceous by at least two global products across two years were removed (for the filtering rules details, see Table S3). This process removed 75,129 points (*i.e*. about 3% of the total), improving the overall quality of our training data (specifically for augmented samples with crop-grassland rotation period less than 5 years) and resulting in 2,353,785 point samples distributed across the time series 2000–2022 (see Figs. S2 and S3).

### GLAD Landsat ARD-2

The primary EO data input for our spatiotemporal modeling was the global Landsat Analysis Ready Data developed by the Global Land Analysis and Discovery Lab at the University of Maryland (GLAD ARD)0^19^. GLAD ARD provides a 16-day time series of tiled Landsat normalized surface reflectance from 1997 onward. The entire Landsat 5, 7, 8, and 9 Collection 2 USGS data archive was used to produce the data set^28^. The Landsat data processing algorithm included per-pixel observation quality assessment, reflectance normalization, and anisotropy correction. The Moderate Resolution Imaging Spectroradiometer (MODIS) MOD44C surface reflectance product was used as a normalization target for a single-step reflectance bias and anisotropy correction. Each 16-day composite includes the best quality observation and contains eight spectral bands (*i.e*. blue, green, red, Near-infrared–NIR, Short-wave infrared 1–SWIR1, Short-wave infrared 2–SWIR2, and thermal) and a quality assessment band that flags clouds, cloud shadows, snow/ice, haze, water, and clear-sky land. Since our reference samples are sparsely distributed over time, we decided to use GLAD ARD instead of the USGS Landsat collection to take advantage of the consistent pixel values across different Landsat systems over the years, improving the temporal generalization of our models and reducing the need of sampling all mapped periods.

### Landsat temporal aggregation and imputation

To reduce the impact of cloud cover and enable the incorporation of intra-annual seasonality in our features, we aggregated the Landsat ARD-2 time series (1997–2022) in bi-monthly temporal composites. For every GLAD tile (*i.e*. 1 × 1 geographic degree), we executed the following steps^29^:

1. Removal of all pixels classified as cloud, cloud shadow, haze, cloud buffer, shadow buffer and shadow high likelihood according to quality assessment band (mask values: 3,4,7,8,9,10);
2. Conversion of pixel values to 8-bit by linear normalization, resulting in values ranging from 0 to 250;
3. Temporal aggregation of all clear-sky pixels for a 2-month period using a weighted average by cloud_cover (estimated for each date and tile);
4. The remaining data gaps were imputed using time-series reconstruction, relying solely on clear-sky pixels acquired on previous dates (*e.g*. gaps in Jan–Feb, 2002 composite considered clear-sky pixels of 1997, 1998, 1999, 2000 and 2001). The imputed values were derived using Seasonally Weighted Average Generalization (SWAG), which applied a vector of weights that prioritized pixel values from the same bi-month period and previous years over those from neighboring regions or different bi-month periods^29^.

### Landsat-derived indices

In addition to the bi-monthly aggregates for the reflectance bands, we also incorporated several key vegetation and water indices as predictor variables for modeling purposes. These indices include the Bare Soil Index (BSI)^30^, Enhanced Vegetation Index (EVI)^31^, the Modified Normalized Burn Ratio (NBR2), also called Normalized Difference Tillage Index (NDTI)^32^, the Normalized Difference Vegetation Index (NDVI)^33^, the Normalized Difference Water Index (NDWI)^34^ and the near-infrared reflectance of vegetation (NIRv)^35^. Each of these indices was derived from different linear combinations of the reflectance bands and provides unique information on vegetation health, moisture content, severity of burns, and overall ecological conditions. We also included a temporal aggregated index, Bare Soil Fraction (BSF)^36^, which is used to capture processes that require a longer temporal frame for sensible quantification: it is determined by the proportion of time the NDVI is <0.35 over the six bi-monthly aggregates^29^. In addition to spectral indices, we derived per-pixel Fraction of Absorbed Photosynthetically Active Radiation (FAPAR) using its correlation with NDVI^37^. Table S4 summarizes the formulas for each Landsat-derived index utilized in our modeling.

### Atmospheric and land surface data

Land surface data was obtained from the MODIS Land Surface Temperature and Emissivity (LST&E) product, specifically MOD11A2^20^. This product is available at a spatial resolution of 1 km and provides 8-day composite data that include both daytime and nighttime surface temperatures. To adapt these data for our analysis, we aggregated the 8-day composites into monthly averages, facilitating the calculation of long-term temperature trends for the period from 2000 to 2022. Specifically, we computed the median (50th quantile) and the standard deviation for both daytime and nighttime temperatures on a monthly basis. This processing yielded a total of 48 input features for our modelling. We also used MODIS water vapor data, specifically the atmospheric product MCD19A2, which captures column water vapour above the ground using near-IR bands. We aggregated the daily product into monthly composites, calculating the mean and standard deviation of positive, non-cloudy observations. The remaining no-data values were imputed using a gap-filling algorithm; for more detailed information on the methodology and data processing steps, refer to the Zenodo entry Parente *et al*.^38^, and Consoli *et al*.^29^.

### Static raster datasets

The elevation data utilized in the modeling was obtained from the Ensemble Digital Terrain Model (EDTM) of the world at 30 m spatial resolution^39^. This DTM results from integrating multiple sources, including ALOS AW3D^40^, GLO-30^41^, MERIT DEM^42^, and various national DTMs. To quantify the isolation from urban areas and correlate it with the livestock management practices, we used a suite of 10 global accessibility indicators calculated at 1 km resolution^43^; class 1 represents areas with travel times of less than 30 minutes to the nearest city of 50,000 or more inhabitants, indicating high accessibility, while class 9 refers to areas where travel time exceeds 10 hours to reach the nearest city of 50,000 or more inhabitants, indicating very low accessibility.

We also independently developed distance maps from permanent or seasonal inland water at 100 m resolution using a Landsat-derived product specifically developed for inland waters^44^. Similarly, we produced maps of distances to areas classified by road density, ranging from low to high, utilizing OpenStreetMap (OSM) data. We also calculated the geometric minimum and maximum temperature as geometric transformations based on latitude, day of the year, and elevation^45^. This calculation considered both the minimum and maximum temperature per month, resulting in 24 input features. These variables not only capture Earth’s geometry and temporal dynamics within a year but also enable the model to differentiate between locations that, despite having similar long-term or monthly temperature profiles, are distinct in their latitudinal positions or seasonal timing. This approach improves the model’s ability to discern and predict on the basis of subtle climatic variations influenced by geographical and temporal factors.

### Spatiotemporal model training

We modeled the grassland classes separately, training one model specialized in cultivated (*i.e*. binary classifier of *cultivated grassland* vs *other land cover*) and another model specialized in natural/semi-natural grassland (*i.e*. binary classifier of *natural/semi-natural grassland* vs *other land cover*). For each model, we ran a feature selection (*i.e*. Recursive Feature Elimination–RFE^46^), a hyperparameter tuning (*i.e*. Successive Halving^47^) and a comparison between three ML algorithms (*i.e*. Random Forest - RF^48^, Gradient-boosted trees–GBT^49^ and Artificial Neural Network–ANN^50^). The modeling strategy used all samples, with different reference years (see Fig. S2), to train a single model able to generalize in time and produce predictions for all years (effective relying in the harmonized Landsat ARD-2 composites).

Before modeling, we overlaid our point samples with the temporal and static EO data. The Landsat pixel values were associated with each sample by spacetime overlay, matching the location (*i.e*. geographical coordinates) and the time period (*i.e*. year of reference) of each sample with 84 Landsat composites in a specific year (*i.e*. seven reflectance bands and seven spectral indices for six bi-monthly aggregates). All samples were treated individually and were associated with the temporal features considering only the year of reference, established by our labeling process. For static layers (*i.e*. long-term MOD11A2 land surface temperature, long-term MCD19A2 water vapor, geometric temperature, static DTM, and static distance maps of cities, roads, and water), the overlay considered only the sample locations, resulting in a total of 197 input features for feature selection. The overlaid samples were then split into training and calibration, where 10% of samples from each visually interpreted tile (*i.e*. 11 km) were randomly selected to compose the calibration set, resulting in 2,122,357 and 231,428 samples for training and calibration, respectively. The calibration set was used to run the Recursive Feature Elimination and then Successive Halving, thus establishing the best features and hyperparameters to compare the ML algorithms.

Our Recursive Feature Elimination^46^ considered a standard Random Forest model with 60 trees and default hyper-parameters (fitted using scikit-learn^51^), targeting 75 features as final selection (*i.e*. about 38% of the total number of features) and removing the four least important features per iteration (according to gini importance). The best 75 features of each model, shown in Table S5, were then used to run Successive Halving, which considered the log_loss metric^22^ and five-fold spatial blocking cross-validation (based on visually interpreted tiles–*i.e*. 11 km) for assessing iteratively different combinations of hyper-parameters candidates bounded by a customized search space. Our Successive Halving started with 500 samples, selecting the best candidates (*i.e*. dropping half of the less accurate candidates) and doubling the number of samples per iteration until reaching the full set of calibration samples. After the last iteration, the hyper-parameters with best log_loss (*i.e*. lowest value), shown in Table S6, were selected for each ML algorithm.

The comparison used the training set and the five-fold spatial blocking cross-validation to estimate accuracy metrics adequate for probability output (*i.e*. R2logloss^52^ and precision-recall curves^53^) for Random Forest, Gradient-boosted trees and Artificial Neural Network. For each algorithm, five ML models were trained using 80% of samples (*i.e*. one fold) and 20% for validation in each iteration, resulting in an out-of-the-fold prediction for all samples. The blocking strategy kept all samples from the same tile (*i.e*. 11 km) either in training or validation set, reducing the spatial correlation between boFth sets and allowing for a more strict evaluation of the error estimate^54^. This analysis excluded the interpolated point samples. The best model according to R2logloss (*i.e*. highest value) was used to train two global models considering all points samples (*i.e*. 2,353,785 samples) and 102 features (*i.e*. union of the best-selected features–see Table S5). The global models were then used to predict (worldwide) *cultivated* and *natural/semi-natural grassland* for all years of the time series.

### Spatiotemporal prediction

Global predictions were produced per GLAD tile (*i.e*. 11 geographic degree) and on a yearly basis from 2000 to 2022, resulting in annual per-pixel probabilities for each class of grassland at 30 m spatial resolution. In an effort to speed up this process, we did not predict pixels mapped as deserts, stable tree cover, salt pan wetlands, stable snow and ocean water in all years between 2000–2020, according to the UMD GLAD GLCLUC product (for a complete list of land cover classes see Table S7). Furthermore, we also excluded areas mapped as buildings by the World Settlement Footprint in 2019, and by the evolution product, which covers every 5 years between 1990 and 2015^55^.

Our Random Forest models were compiled to a native C binary using TL2cgen^56^, reducing the prediction time by factor 3. After running the predictions, the time-series of probabilities were smoothed out by a spatio-temporal filter, which considered a three-dimensional Savitzky-golay–SG (polynomial order three and squared window with five pixels) to reduce the inter-annual variability in the prediction outputs. Savitzky-golay is a robust filter capable of significantly reducing local noise/spikes without changing the main trend of the time-series^57^. Additionally, we produced a Mean Absolute Difference (MADi) layer for each class of grassland, where we estimated the absolute difference between the predicted and the smothered probabilities and aggregated all years by average.

All these processing steps ran on a High-Performance Computing (HPC) infrastructure and were distributed among the processing nodes using SLURM^58^ and Docker containers^59^. Approximately 120,960 CPU hours and 7.2 terabytes of RAM were used to produce the final predictions. All predicted tiles were then used to create Cloud-Optimized GeoTIFF (COG) mosaics and made publicly available in Google Earth Engine and the SpatioTemporal Asset Catalog (STAC).

### Dominant grassland production

The cultivated and natural/semi-natural grassland probabilities (smoothed with Savitzky-golay) were used to derive annual dominant grassland maps based in a customized probability threshold. For each class, we calculate the precision-recall curves^53^ through five-fold spatial blocking cross-validation and using 2,1 million points samples. The curves were then used to find which probability threshold provides balanced/equal recall (*i.e*. producer’s accuracy) and precision (*i.e*. user’s accuracy). All probabilities greater or equal to the selected thresholds were converted to dominant grassland classes. For pixels classified simultaneously as dominant in our two grassland classes, we kept only the class with the higher f1-score according to our cross-validation strategy (*i.e*. natural/semi-natural grassland).

## Data Records

**Fig. 3:**
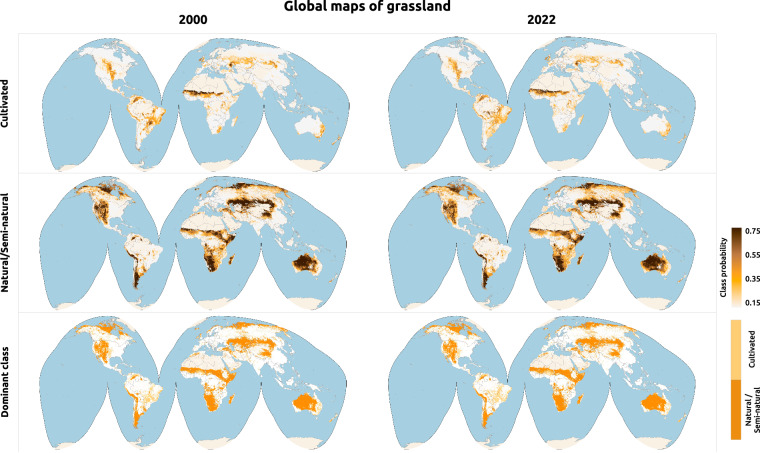
Global grassland maps for 2000 and 2022 including dominant class and probabilities for cultivated and natural/semi-natural grassland.

The global grassland maps described in this paper are available from 2000–2022 in COG (Cloud Optimized GeoTIFF) format under the Creative Commons license CC-BY, archived in Zenodo (10.5281/zenodo.13890401^60^ - Fig. 3), and publicly accessible in OpenLandMap SpatioTemporal Asset Catalog (STAC - https://stac.openlandmap.org/gpw_ggc-30m/collection.json). The COG format supports HTTP range requests, enabling seamless lazy loading access by GIS solutions (*e.g*. Quantum GIS, MapServer, GeoServer, etc) and programming environments (*e.g*. JupyterLab, RStudio, etc).

A total of 69 global mosaics (*i.e*. 23 years for each time series) is available in the WGS84 Coordinate Systems (*i.e*. EPSG:4326) and pixel size equal to 0.00025 degrees. The grassland probability values range from 0–100, and the class values used by the dominant maps are zero (0) for *other land cover*, one (1) for to *cultivated grassland* and two (2) for *natural/semi-natural grassland*. All raster files are in unsigned 8-bit integer format and use 255 as no-data value (pixels which were ignored in by predictions according to the UMD GLAD GLCLUC product; see Table S7), following a naming convention that organizes the most important data properties in nine fields:

1. Project name: Global Pasture Watch (gpw)
2. Class name: *cultivated grassland* (cultiv.grassland), *natural/semi-natural grassland* (nat.semi.grassland) and dominant grassland (grassland)
3. Procedure combination: Random Forest (rf), Savitzky-golay (savgol), balanced threshold (bthr) and mean absolute difference (madi).
4. Variable type: probability (p) and class (c)
5. Spatial resolution: 30 m
6. Begin of time reference: date of first Landsat composite used by the modeling (20220101)
7. End of time reference: date of last Landsat composite used by the modeling (20221231)
8. Spatial extent: global (go)
9. Coordinate system: World Geodetic System 1984, used in GPS (epsg.4326)
10. Version: v1

## Technical Validation

### Spatial cross-validation and feature importance

Our comparison results, shown in Table 3, revealed very similar R^2^_logloss_ values for tree-based algorithms (*i.e*. Random Forest and Gradient-boosted trees), while Artificial Neural Network presented the lowest values for both classes of grasslands. We used the precision-recall curves to define probability thresholds that can balance precision and recall (*i.e*. similar values) and maximize the F1 score^53^. Artificial Neural Network had the highest probability threshold, while Gradient-boosted trees had the lowest one. These thresholds were used to convert probabilities in dominant classes (*e.g*. all samples with predicted probabilities greater than or equal to 0.32 were converted to *“Cultivated grassland”* class), which were then used to estimate the F1 score. Gradient-boosted trees presented F1 scores slightly higher than Random Forest, and Artificial Neural Network presented the lowest scores for both grass classes. As there were no significant differences in accuracy between Random Forest and Gradient-boosted trees, we decided to use Random Forest to train the final global models due to the speed-up possibility offered by TL2cgen^56^.

**Table 3:** Comparison of ML algorithms derived by five-fold spatial blocking cross-validation using 2,122,357 points samples.

ML algorithm	Cultivated grassland			Natural/Semi-natural grassland		
	R^2^_logloss_	Balanced probability threshold	F1 score	R^2^_logloss_	Balanced probability threshold	F1 score
Random Forest - RF	0.924	0.328	0.644	0.773	0.428	0.759
Gradient boosting trees - GBT	0.924	0.162	0.653	0.767	0.352	0.760
Artificial Neural Network - ANN	0.916	0.380	0.607	0.697	0.468	0.720

The probability thresholds were defined based on a precision-recall curve aiming to maximise the F1 score.

The accuracy matrix, derived using the probability thresholds shown in Table 3, presented higher accuracies for *natural/semi-natural grassland* than *cultivated grassland* (see Table 4). The class *other land cover* had values greater than 0.90 in all accuracy metrics. In addition to the massive number of points samples and robustness of the spatial blocking cross-validation^54,61^ and sampling design (*i.e*. FSCS), the current accuracy was based on 7,005 tiles where we had VHR imagery available for the labeling process. Tiles without reference labels might have very specific grassland dynamics that have not been captured by our models and accuracy assessment. Furthermore, our reference data are quite sparse in time, with 40% of tiles having a single year available for visual interpretation, and most of the samples obtained in 2009–2014 and 2019–2022 for Bing and Google Maps, respectively (see Fig. S3). This temporal sparsity makes inferences based on sample-based annual areas currently not possible for our grassland classes, even that considering all years, the proportion of *cultivated grassland* and *natural/semi-natural grassland* together reaches 32% (see Fig. S2).

**Table 4:** Accuracy matrix for the final Random Forest models estimated by five-fold spatial blocking cross-validation using 2,122,357 points samples.

	Expected				Recall (Producer’s acc.)
Predicted		Cultivated grassland	Other LC	Total	
	Cultivated grassland	**0.062**	0.034	0.096	0.643
	Other LC	0.034	**0.869**	0.904	0.962
	Total	0.096	0.904	**1.000**	
Precision (User’s acc.)		0.644	0.962		
	**Expected**				**Recall (Producer’s acc.)**
		Natural/Semi-natural grassland	Other LC	Total	
Predicted	Natural/Semi-natural grass	**0.202**	0.064	0.266	0.758
	Other LC	0.064	**0.670**	0.734	0.913
	Total	0.266	0.734	**1.000**	
Precision (User’s acc.)		0.759	0.913		

The precision and recall were balanced considering the probability threshold 0.32 and 0.42 for *cultivated grassland* and *natural / semi-natural grass*, respectively.

To overcome these issues, work is ongoing to independently validate output layers (led by IIASA) based on a new set of reference samples and a different group of analysts, following the good practices of evaluation for LULC products^62^ and able to support a proper assessment of grassland land cover changes/dynamics. Visual interpretation has been conducted on the Geo-Wiki platform considering the current class definitions/criteria and multiple satellite imagery to address the temporal sparsity (*e.g*. Google Maps, Bing Maps, Landsat and Sentinel)^63^. This validation helps assess and measure concrete improvements in the next versions of grassland maps since we can reinterpret our current training samples based on feedback and local knowledge without changing the independent validation samples. Additionally, we will evaluate the quality of our cross-validation assessment, measuring how well our ML models will perform on a new set of reference samples.

**Fig. 4:**
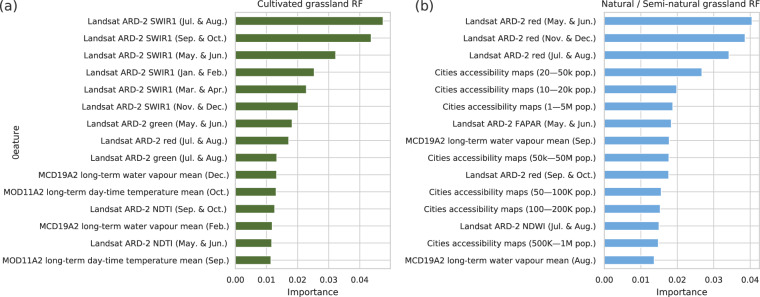
Top-15 most important features according to our global Random Forest (RF) models for: (**a**) cultivated grassland, and (**b**) natural/semi-natural grassland.

Feature importance of our Random Forest models shows that SWIR1 is the most important Landsat band for identifying *cultivated grassland*, with the highest importance for all bi-monthly periods (see Fig. 4a). The green and red bands, together with NDTI (Normalized Difference tillage Index), are also important Landsat features and probably contribute to the distinction of *cultivated grassland* and croplands. The long-term MODIS water vapor (December and February) and the MODIS daytime temperature (October and September) are the only coarser resolution layers (*i.e*. 1 km) among the top-15 most important features. For *natural/semi-natural grassland*, eight of the 15 features are coarser resolution layers, including several city accessibility maps^43^, which are probably contributing to the identification of remote grassland areas (*e.g*. nature reserves, semi-arid grasslands, tundra ecosystems). Nevertheless, red is the most important Landsat band for distinguishing this class of grasslands, specifically the May to December (*i.e*. four bi-monthly periods–see Fig. 4b) seem to help the predictive mapping especially.

### Independent validation with existing samples

To comprehensively compare our global grassland maps with existing LULC mapping initiatives, we harmonized reference samples from 7 datasets, shown in Table 5. This process involved translating the original LULC classifications of these datasets into our three classes (*i.e. grassland*, *natural/semi-natural grassland* and *other land cover*), leveraging the original class definitions and expert knowledge to map LULC across different datasets accurately. This involved meticulously comparing the definitions of LULC classes within each dataset with the classification scheme described above. The crosswalk/class harmonization tables were implemented using Python computational notebooks and are available in Zenodo^64^. As a result, we obtained 66,991,467 harmonized individual samples (unique points in geographical space and time - 10.5281/zenodo.13951976^64^).

**Table 5:** Datasets of pre-existing reference samples harmonized to our classification taxonomy.

Datasets	Original license	Spatial distribution	Time period	Number of individual samples
WorldCereal^70^ (10.5281/zenodo.7593734)	CC-BY-4.0	Global	2016–2021	36,427,760
EuroCrops^89^ (https://zenodo.org/records/10118572)	CC-SA-4.0	Europe	2018–2021	13,484,591
MapBiomas Brazil^17^ (10.5281/zenodo.5136666)	CC-BY-4.0	Brazil	2000–2018	1,103,003
GLanCE^18^ (10.34911/rdnt.x4xfh3)	CC-BY-4.0	Global	2000–2021	8,374,634
LUCAS *in-situ* LCLU data^67^ (10.6084/m9.figshare.9962765.v2)	CC-BY-4.0	Europe	2006–2018	989,892
LCMAP CONUS Reference Data^90^ (10.5066/P933Z1TK)	Public Domain	U.S. (CONUS)	2000–2018	341,943
CGLS-LC training dataset^91^ (In preparation for pub.)	CC-BY-4.0	Global	2021	8,269,554
Total				66,991,467

**Fig. 5:**
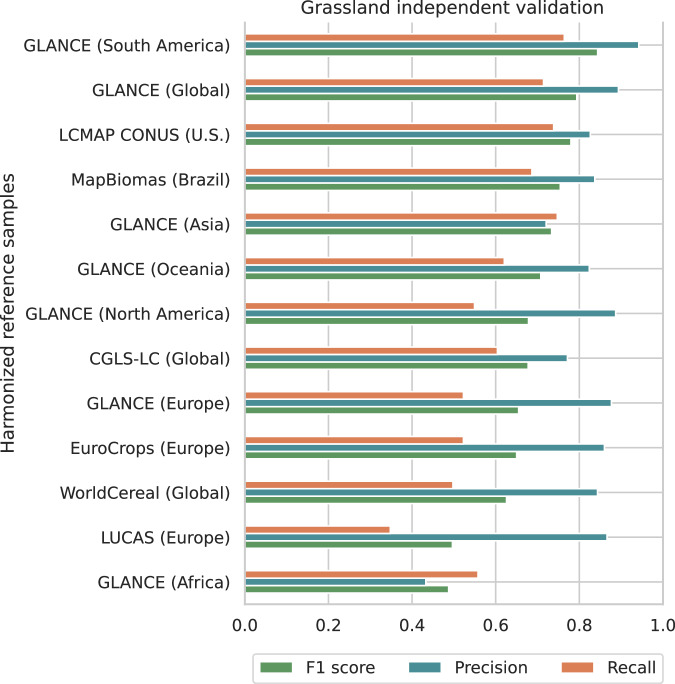
Independent validation of grassland class (*i.e*. cultivated and natural/semi-natural grassland combined) based on harmonized existing reference datasets and and sorted ascending by F1 score.

The harmonized samples were used in to conduct an independent validation of the dominant grassland-class maps (cultivated and natural/semi-natural combined - Fig. 3). This analyses revealed higher precision (*i.e*. user’s accuracy) than recall (producer’s accuracy) in all datasets (see Fig. 5), indicating, in general, that our grassland predictions are more conservative and might not include regions defined as grassland/shrubs by multiple LULC mapping initiatives. Globally, our dominant class maps have precision values higher than 0.7 and F1 scores of 0.79, 0.65 and 0.63 according to GLanCE, CGLS-LC and WorldCereal, respectively.

Specifically for GLanCE, the accuracy metrics were derived per continent, enabling cross-checking with continental and national datasets. F1 score values greater than 0.8 were found for South America (GLanCE) and Brazil (MapBiomas), a key agricultural frontier with the historical expansion of cultivated grassland^65^. Higher accuracy values were found for the U.S. (LCMAP CONUS) compared to North America, indicating more accurate predictions for the country in relation to the rest of the continent. Oceania had similar accuracy values compared to North America, which may be explained by similar patterns in their land cover footprint^66^. Asia presented the most balanced precision and recall among all continents, remarkably similar to our cross-validation values (3). In Europe, the F1 score was 0.64, 0.63 and 0.50 according to GLanCE, EuroCrops and LUCAS, respectively, indicating less accurate predictions compared to other continents, with systematic omission error (recall between 0.35 and 0.53). The low accuracy values obtained with LUCAS might indicate significant mismatches between grassland classification taxonomies^67^. The lowest accuracy values were obtained in Africa, and it is probably related to the widespread disagreement among existing LULC datasets in the continent^68^.

**Fig. 6:**
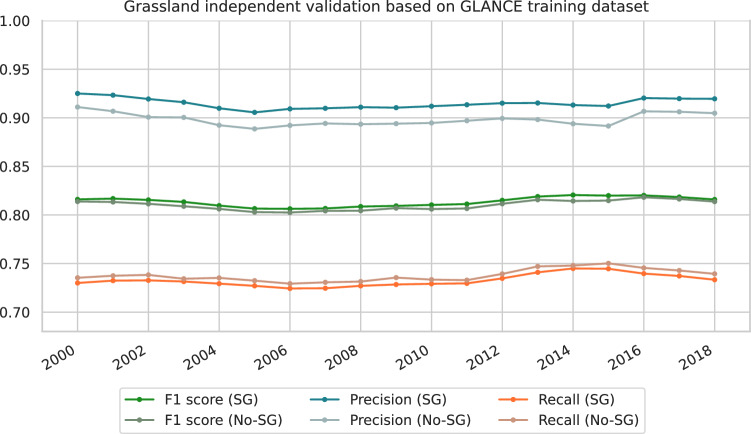
Independent validation of grassland class (*i.e*. cultivated and natural/semi-natural grassland combined) based on GLANCE training dataset. The GLANCE classes grassland (12), shrub (10) and moss/lichen (13) were reclassified to grassland for matching with our legend. All metrics were derived for smoothed probabilities (*i.e*. Savitzky-golay - SG) and non-smoothed (*i.e*. No-SG) considering balanced thresholds of 0.32 and 0.42 for cultivated and natural/semi-natural grassland, respectively.

Considering the wide temporal coverage of GLanCE, we used it to conduct an annual independent validation of our dominant class maps. Since its temporal distribution is not regular across the time series (with several samples having class labels for one to three years), this analyze considered only samples with 10 or more years labeled between 2000–2018. We notice a minor increase in precision (*i.e*. 0.9394 and 0.931 on average for smoothed and non-smoothed probabilities, respectively) followed by a minor decrease in recall (*i.e*. 0.7410 and 0.7449 in average for smoothed and non-smoothed probabilities, respectively) due to SG (Fig. 6). Combined with a visual assessment of probabilities, this confirms that SG increases the spatiotemporal consistency of our predictions without significantly changing their accuracy. The accuracy metrics remain stable throughout the years and show higher precision (*i.e*. user’s accuracy) than recall (producer’s accuracy) across all years, revealing a systematic omission error (*i.e*. false negatives), rather than a commission error (*i.e*. false positives). This can be partially attributed to the establishment of balanced probability thresholds independently for each class, which does not ensure comparable precision and recall values for the combined classes. Compared to the naive threshold, on the other hand, (*i.e*. 0.5) the balanced thresholds increased the F1 score by 0.1241 and recall by 0.1892, on average, while decreased the precision by 0.0369, on average (see Fig. S4).

**Fig. 7:**
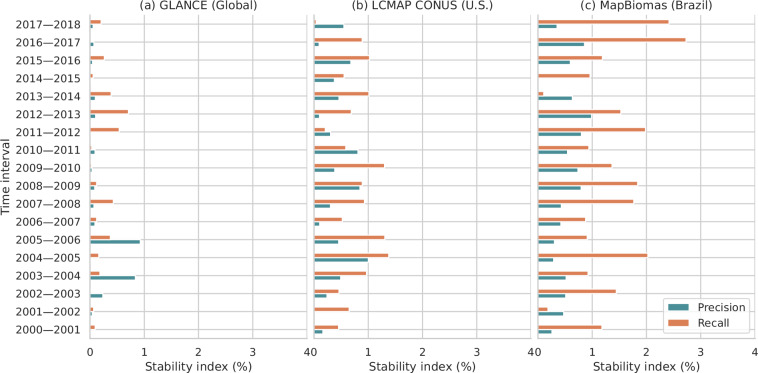
Stability index^69^ estimated for grassland (*i.e*. cultivated and natural/semi-natural grassland combined) based on GLANCE (**a**), LCMAP CONUS (**b**) and MapBiomas (**c**), presenting the absolute percentage difference in precision and recall metrics for two consecutive years.

Aiming to evaluate the temporal consistency of our grassland maps, we estimated the stability index for precision and recall^69^ from 2000 to 2018 using GLanCE, MapBiomas and LCMAP CONUS (see Fig. 7). Stability index is basically the absolute percentage difference of a specific accuracy metric between two neighborhood years, where values close to zero indicate more stable predictions. At global scale (GLANCE), the averaged stability index is 0.15 and 0.21 for precision and recall, respectively. In U.S. (LCMAP CONUS) and Brazil (MapBiomas) the stability index is higher, with averaged values of 0.41 and 0.53 for precision, and 0.77 and 1.35 for recall, respectively for each country. Considering that the grasslands are quite dynamic in the two countries, our predictions are probably not matching in time with the reference samples, and some of the grassland conversions are captured a few years later or completely missed in the time-series.

### Comparison with other LULC maps

To complement our independent validation, we performed a spatial comparison between the grassland maps and 30 m global land cover products, UMD GLAD GLCLUC^13^ and the GLC_FC30^15^, respectively. For each grassland class (*i.e*. cultivated and natural/semi-natural), we calculated the overlap with LULC classes from the products for 3 years (2000, 2010 and 2020). To allow for easier comparison, we combined some of the classes (deciduous and broadleaf forest into a *Forest* class, for example) in each of the LULC products and additionally combined any classes with less than 3% overlap with the grassland classes into the *other* class. With this comparison, we want to identify potential confusion between our grassland predictions and unexpected LULC classes. For example, we expect our *grassland* classes to overlap with the *grassland* class from GLC_FC30 rather than the *forest* class. The comparisons revealed that the grassland proportions do not change over time, so we show only three years out of 20.

Comparison between UMD GLAD GLCLUC and our grassland classes revealed that most of the overlap occurs with the *short vegetation* class (71% for cultivated and 78% for natural/semi-natural), with *croplands* (16% for cultivated) and with *wet short vegetation* (16% for natural/semi-natural). Confusion between cultivated grassland and croplands is expected, as these classes may have very similar spectral-temporal responses in EO imagery^70,71^) and overlapping taxonomies (*e.g*. hay is a type of grass that is planted but falls outside our definition of cultivated grasslands). The comparison between GLC_FC30 and our grassland classes revealed that most of the overlap occurs with *grasslands* (24% for cultivated and 27% for natural/semi-natural), *rainfed cropland* (21% for cultivated), *herbaceous cover cropland* (27% for cultivated), *shrubland* (11% for cultivated and 22% for natural/semi-natural), and *sparse vegetation* (21% for natural/semi-natural). There was unexpected overlap between grassland and *forest* (14% for cultivated and 12% for natural/semi-natural).

**Fig. 8:**
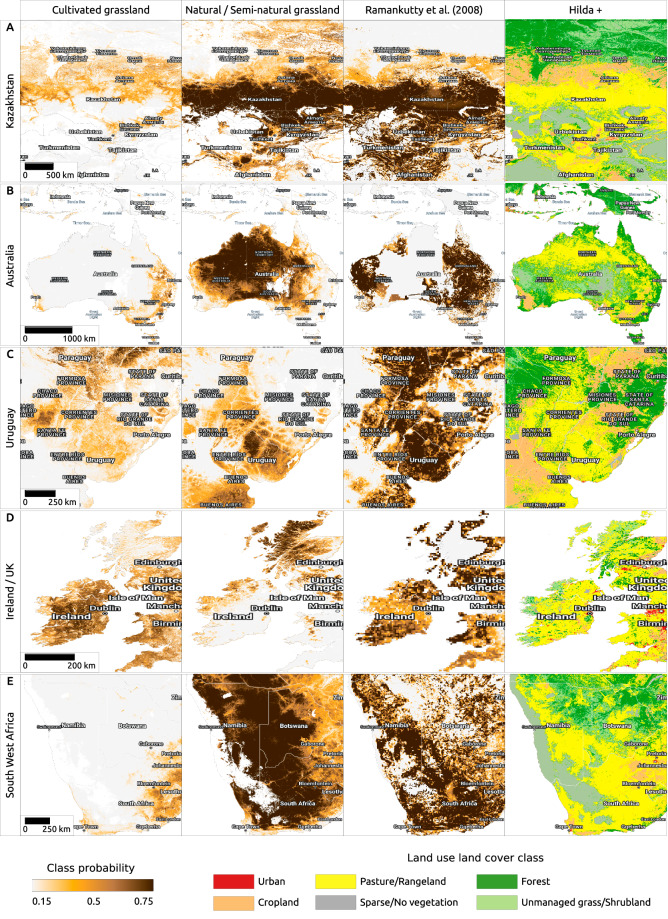
Comparision of pastureland distribution map produced by Ramankutty *et al*.^9^, land cover classes at 1 km resolution based on HILDA+ data set^10^, and our predictions for cultivated and natural / semi-natural grassland at 30 m resolution focused in: (**A**) Kazakhstan, (**B**) Australia, (**C**) Uruguay, (**D**) Ireland / UK and (**E**) South West Africa.

However, comparison between our predictions and 30 m products time-series of land cover is limited because our grassland classes are defined based on the use and overlap of 3 + classes (*e.g. grassland, shrubland, short vegetation*) in either of the two LULC legends. The only global grassland products we can compare with our predictions are coarse resolution, such the 10 km pasture map of the world for the year 2000^9^ and the HILDA+ distribution of *pasture/rangeland* and *unmanaged grass/shrubland* at 1 km resolution^10^ (see Fig. 8). Comparing our predictions of *cultivated grassland*, in general, shows a good match, especially with the global pastureland map by Ramankutty *et al*.^9^; when looking more closely, it seems that the previous products miss some smaller patches where we are certain they can be classified as pastures, but were probably difficult to distinguish from other cropland similar to them or were just too small for resolution of 1 km.

A comparison between HILDA+ and our grassland predictions reveals similar patterns of overlap as described above; however, in this case, we also wanted to assess whether there are grassland areas that we are missing (as demonstrated by the accuracy assessment based on the GLANCE training dataset) and found that 11% and 12% of our *other land cover* class fall within areas classified in HILDA+ as *pasture/rangeland* and *unmanaged grass/shrubland*, respectively. Moreover, 6% of our *other land cover* class falls within the pasture class for the year 2000 of Ramankutty *et al*.^9^ map. While some of this overlap can be explained by the difference in spatial resolution between the two products (30 m vs 10 km), some of it is due to the under-prediction of the extent of grasslands in our product. On the other hand, because our analysis is not limited to pasturelands, the extent of our natural grasslands far exceeds the extent of pasturelands as reported by Ramankutty *et al*.^9^.

## Usage Notes

Users can provide feedback and report classification errors for dominant class maps in Geo-Wiki and all the maps (4 terabytes in total) are also publicly accessible in the follow platforms:

- Geo-Wiki (Feedback tool): https://geo-wiki.org
- Google Earth Engine Apps:
  - Map customization: https://global-pasture-watch.projects.earthengine.app/view/ggc-30m
  - Comparison tool: https://ee-vieiramesquita.projects.earthengine.app/view/ggc-30m-comparison
- Earth Engine Image Collections:
  - projects/global-pasture-watch/assets/ggc-30m/v1/cultiv-grassland_p
  - projects/global-pasture-watch/assets/ggc-30m/v1/grassland_c
  - projects/global-pasture-watch/assets/ggc-30m/v1/nat-semi-grassland_p

### Grassland probability maps

**Fig. 9:**
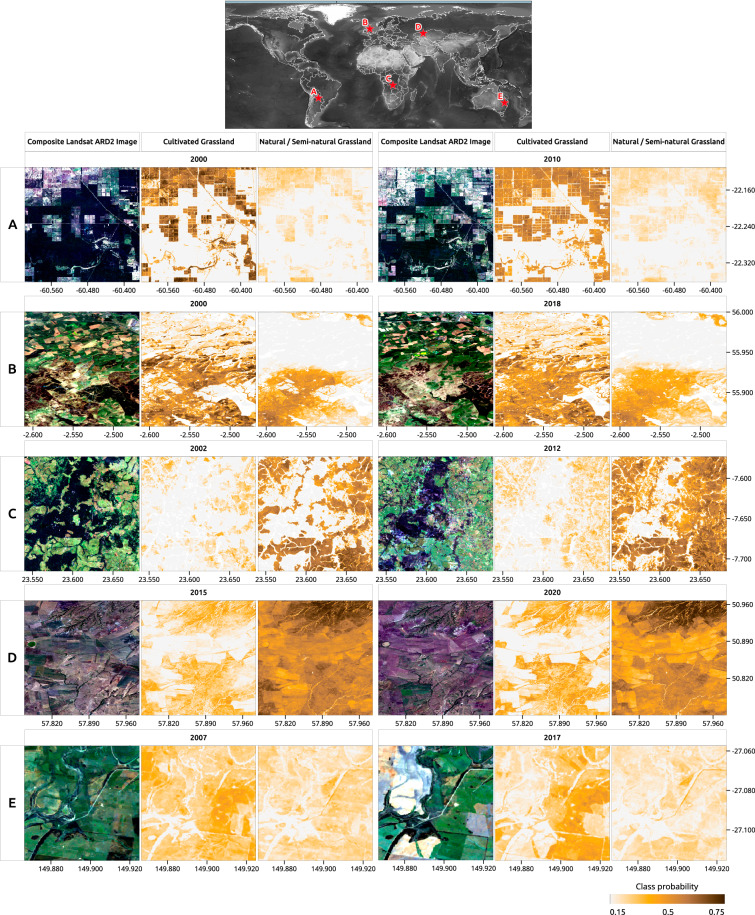
Examples of predicted probabilities for cultivated and natural/semi-natural grassland in (**A)** Paraguay, (**B)** Scotland - UK, (**C)** Democratic Republic of the Congo–DRC, (**D)** Kazakhstan and (**E)** Australia. Landsat ARD-2 images are shown as true colour composite (red, green and blue) for the year of grassland probabilities. The composites are from Mar. & Apr. (all years) in Paraguay and Scotland; Mar. & Apr. 2002 and Nov. & Dec. 2012 in DRC; Aug. & Sep. 2015 and May. & Jun. 2020 in Kazakhstan; and May & Jun. 2007 and Mar. & Apr 2017 in Australia.

**Fig. 10:**
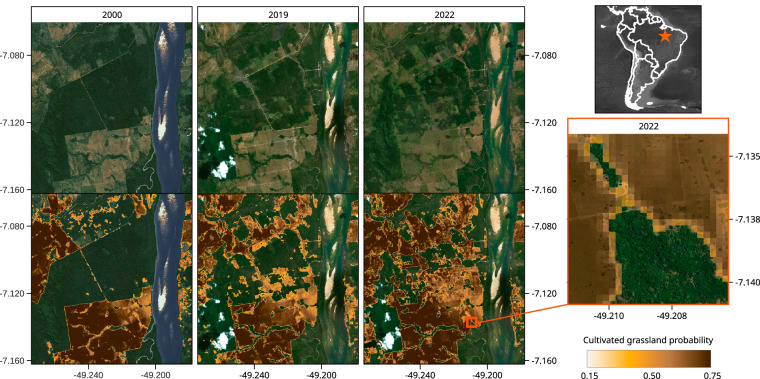
Cultivated grassland probabilities for 2000, 2019 and 2022 at 30 m spatial resolution (below) for a deforested area in in Brazil (Rio Maria, Pará state) as compared to the very high resolution images of ESRI Wayback (above).

The main data output described in this paper is the time series of probabilities for two classes of grasslands (*i.e*. cultivated and natural/semi-natural representing the end members of a spectrum of grassland definitions, selected primarily based on the capacity of identifying them in VHR imagery), estimated independently by global Random Forest models. In general, our predictions are able to capture the expansion of cultivated grassland over different types of native vegetation in tropics (see Figs. 9a,c and 10), and distinguish between grassland and cropland in, for example; Europe (Fig. 9b), Asia (Fig. 9d) and Australia (Fig. 9e) over multiple years.

Global modeling enables custom thresholds for converting probability values into dominant classes seamlessly and consistently, once all pixels are predicted using the same model for all years across the world. To demonstrate this application, we derived global maps for dominant classes considering balanced probability thresholds, where precision and recall have similar values according to our five-fold spatial blocking cross-validation (*i.e*. 0.38 for *Cultivated grassland* and 0.42 for *Natural/Semi-natural grassland*), resulting in more area mapped as grassland (both classes combined) compared to a naive threshold (*i.e*. 0.5–see Fig. S4). However, the assessment with existing independent reference sample datasets consistently showed greater precision than recall (*i.e*. more omission than commission error for dominant classes), which can be partly explained by the inherent limitations in harmonizing multiple grassland definitions with our classification taxonomy. The independent accuracy assessment paired with the visual comparison with existing land cover products have shown that, most likely, the maps for dominant classes are providing a conservative estimate for global grassland areas. Users of dominant class maps should additionally note that our global thresholds were derived from ∼70% of total tiles (*i.e*. 1 × 1 km) determined by our sampling design and may not cover specific grassland regions where VHR imagery was not available. Additionally, our predictions were based on independent ML models, which treated each class separately and resulted in several grassland areas mapped simultaneously as cultivated and natural/semi-natural after applying the balanced probability threshold (See Fig. 9). As *natural/semi-natural grasslands* reached a higher accuracy than *cultivated grassland*, pixels that reached the required threshold in both classes were assigned the natural/semi-natural class over the cultivated one, which additionally assumes a position in line with the precautionary principle for monitoring global natural/semi-natural grasslands^72^.

**Fig. 11:**
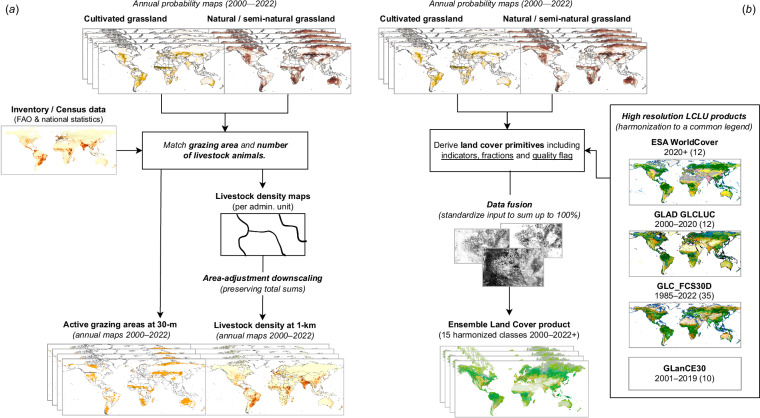
Future Global Pasture Watch applications for the produced grassland probability maps: (**a**) to delineate for example active grazing areas matching with census estimates and help produce more reasonable livestock density maps^75^, (**b**) to help produce global time-series of ensemble land cover products harmonizing and combining multiple existing products (Esa WorldCover^14^ UMD GLAD GLCLUC^13^, GLC FCS30^78^ and GLanCE30^77^).

Our mapping strategy has the main aim of providing probabilities that allow the production of customized maps of dominant grassland classes (as demonstrated in the current study) and empower users to define their own decision and integration rules (*e.g*. probability threshold, class priority, other land cover masks). For example, a user interested in South African grasslands can select a specific probability threshold based on national reference samples, prioritize cultivated over natural/semi-natural grasslands and mask areas mapped as cropland by existing land cover maps. In this way, the global maps provided here constitute an integral component of a broader framework led by GPW focusing on grassland, pastures, and livestock monitoring (see Fig. 11a). Some of the potential uses identified in project conception which are aimed to serve a wide range of organizations and user communities at global, national, and local scale, include the following:

- **Precision-recall calibration**: Reference grassland samples, including *in-situ* data, can be used to estimate precision-recall curves for target areas (*e.g*. watersheds, biomes, administrative areas), enabling the development and use of locally calibrated thresholds. Such local probability thresholds would necessarily differ from those found in our global analysis (*i.e*. 0.32 for *Cultivated grassland* and 0.42 for *Natural/Semi-natural grassland*), and are likely to result in grassland maps which more accurately reflect the target local area. In addition to balancing precision and recall, other criteria could be used to define the threshold, minimizing the error of omission, for example, based on the Murashkin *et al*.^73^ method.
- **Area estimation calibration**: Known or estimated quantities of *cultivated grassland* and *natural/semi-natural grassland* in an administrative area, for example, through reports or census results, can be used to derive thresholds that explicitly enforce correct and spatial class proportions. Recent findings suggest that this can be done in a way that actually modestly improves overall map accuracy, especially in parts of the map where classes are mixed or atypical in the feature space^74^, which might be particularly useful to match grazing areas with livestock census records in the context of the Gridded Livestock of the World product^75^.
- **Land cover primitives**: Combined with other land cover products, probability maps can be used as *“primitives”*/ which are considered as building blocks for the construction of ensemble land cover products (see Fig. 11b). *“Primitives”* represent raw information needed to make decisions within a dichotomous key applied to land cover typologies, and recent findings have shown consistent and promising results through an implementation that assumes Random Forest probabilities as land cover primitives^76^. In addition to probabilities, dominant land cover classes from existing products (*e.g*. GLanCE30^77^, GLC FCS30^78^, MapBiomas^17^) can be used as *“primitives”* if converted to indicators (*i.e*. binary rasters); weighted by expert-based rules and averaged by standardization fractions that sum up 100% amongst all inputs. Although this possibility can take advantage of several land cover products in a holistic and multi-scale way; the process of legend harmonization amongst the classes might constitute an undefined source of uncertainty and requires further investigation.

### Current limitations and mapping feedback

Despite the flexibility provided by the probability maps, we note several classification issues and limitation in our grassland predictions (see Table 6). Most of these issues (*e.g*. specially the miss-classification errors) are not trivial to resolve in the face of Random Forest as a complex and non-linear prediction system, as we are not sure these outcomes happen because of (1) extrapolation problems, (2) noise/limited detectability in the Landsat images, (3) fuzzy definition of grassland classes, (4) need of more specialized and regional/local ML models, or (5) simply a lack of training points in these areas. Our best approach moving forward is to simply increase the representation of regional cultural knowledge in these areas and assess the accuracy of future versions of the maps with global and local reference validation samples/datasets.

**Table 6:** Issues and limitation currently identified in the global grassland maps.

Under-estimation of grassland extent
• Grassland extent is under-predicted in southeastern Africa (mainly in Zimbabwe and Mozambique) and in eastern Australia (mainly in the shrublands and woodlands of the Mulga ecoregion).
**Cropland misclassified as grassland**
• In the state of Montana, USA, cropland areas located on historical prairie areas have high probability values for *natural/semi-natural grassland* and low values for *cultivated grassland*,
• In arid and hyperarid landscapes of northern Africa and the Arabian Peninsula herbaceous croplands areas (irrigated pivot agriculture) presented high probability values for *cultivated grasslands*,
• Sudan, Niger, Uganda, Kenya, and Mali have several cropland areas with high probability values for *natural/semi-natural grassland*,
• In the state of Western Australia, New Zealand, the center of Bolivia, and the state of Mato Grosso (Brazil), large cropland areas have high probability values of *cultivated grasslands*.
**Mixed farmland mosaics misclassified as grassland**
• Farmland mosaics in North-Eastern Uganda’s present high probability for both grassland classes,
• In eastern Madagascar extensive areas of shifting agriculture have high values for cultivated grassland probabilities
**Woody vegetation lost misclassified as grassland**
• In arid and hyperarid landscapes of northern Africa and the Arabian Peninsula mixed crop-livestock systems and tree crops presented high probability values for *cultivated grasslands*,
• Western African Sahel belt, the Northern-Central African and the savanna-desert transition zone (Eastern Chad/Western Sudan) have high values of *cultivated grassland* probabilities in intensively grazed areas with partially lost woody vegetation,
• Non-cultivated (low-input) pastures/herbaceous cover in recently deforested areas in Selva Maya (Chiapas, Petén) and the Arc of Deforestation in Amazon Region presented high probability values for *cultivated grasslands*.
**Macroscopic errors**
• Although important for grassland separation, the 1-km accessibility maps and MODIS products (MOD11A2 and MCD19A2) introduced curvilinear macroscopic errors (due to the downscaling strategy based on cubicspline) in Uruguay, Southwest Argentina, South of Angola and in Sahel region in Africa.
• Due to the Landsat 7 Scan Line Corrector failure, regular stripes of grassland probabilities are visible at parcel-level. This issue is more prominent in 2012, where GLAD Landsat ARD-2 relies only in Landsat 7 imagery.

Nevertheless, we can reasonably assume that some of these issues are related to very similar values of two or more classes in the feature space (limited detectability in Landsat images), where our ML models did not allow separation among areas with distinct LULC dynamics as embodied in our visually interpreted training dataset. It appears that intensively managed grasslands, with high homogeneity under many conditions, have a high chance of being confused with other classes that have very similar spectral properties, such as urban mosaics (*i.e*. buildings, sparse trees and grass fields with different densities) or (greenish) croplands with similar vegetation height and spatial configuration (such as cereal crops^70,71^). Less intensively cultivated grasslands, where more diverse plant species can be found and where the landscape may not be very regular, are easily confused with grasslands that are not cultivated or (semi) natural herbaceous vegetation, in general^68^. In addition, the spectral signal of cultivated grasslands can not be as clearly distinguished from natural/semi-natural grassland as it could be from croplands, where there are clear breaks in vegetation growth in cases where multi-temporal clear-sky images are available^79^.

The distinction between cultivated and natural/semi-natural grasslands has been notoriously difficult to map in the past^16,17,80^, which has also affected our reference data collection and harmonization process. Hence, our reference labeling protocol relied on more indirect indicators of management, such as fences and other typical infrastructure, hay bales, machine presence, and even animal presence in the field or geometric shapes of the landscape. This may lead to an underestimation of signs of cultivation that may be less intensive or where VHR imagery was not available at the time of management practices. Regarding our harmonization process, the description or labeling among different datasets is a limiting factor. Since we analyzed samples from a wide range of sources, all with their own ontological definitions and classification taxonomy, harmonization was possible only based on rough estimations. Even when acknowledging language and conceptual differences; some fundamental differences between scientific domains/schools of thought/cultural views may also result in ambiguous terms or descriptions. For example, while it may be called *“rangeland”* in the U.S., the same concept would be called *“pasture”* in Europe, while a *“pastagem”* (the literal translation of ‘pasture’) would be regarded as a cultivated grassland in Brazil. Often, the finer distinctions of how dataset creators perceive and interpret mental concepts whilst creating the training dataset, is missing from their fundamental description, making it harder for downstream applications to form a proper semantic match across many datasets. Due to these challenges, we have attempted to be as clear and as transparent as possible in our reference labeling criteria and to plan for active inclusion of regional cultural knowledge in further versions of Global Pasture Watch products.

One possible way to resolve such semantic/ontological issues is through international registers where land cover and land use classes/systems are unequivocally specified and illustrated with decision trees and photographs accompanied by multi-lingual descriptions. However, for this, the international community would have not just to provide such context, but to also have to agree on some thresholds and recommendations, such as the minimum livestock densities in relation to productivity, the minimum number of years under some land use system, and the duration of fallow periods. Disregarding such forward looking assertions, our predicted grassland distribution for 2000–2022 aims to become an integral component of a broader framework of monitoring products to be produced by Global Pasture Watch and will also include aspects of grassland productivity, fraction of scrubs and woody vegetation, and densities of multiple livestock animals (*i.e*. cattle, goat, sheep, buffalo and horses). The data set presented here is the first essential step toward these future products, serving as both a pioneering demonstration and a foundation for ongoing refinements (follow the project at https://landcarbonlab.org/data/global-grassland-and-livestock-monitoring/).

Users need to be aware of the limitations and the known issues discussed in this section; whilst considering them carefully to ensure appropriate use of maps at this initial prediction stage (*e.g*. we do not recommend the usage of our global maps as replacement for fieldwork campaigns and/or source of ground-truth data for grassland ecosystems). Alongside noting shortcomings in current maps, we are working actively to address most of the these issues through mapping feedback campaigns on the Geo-Wiki platform, where experts and/or users with local knowledge of LULC classes can visualize and interact with the most recent versions of our products. Additionally, all global products used in our comparison analyzes (UMD GLAD, GLC FCS30D, HILDA+, Ramankutty *et al*.^9^) have been uploaded on the platform, supporting users in the provision of feedback regarding overall agreement, spatio-temporal consistency, and over- and under-estimated grassland extent. Solicited feedback via Geo-Wiki may consist of drawing polygons in designated or non-designated areas, concentrating on the differentiation of (1) grassland or non-grass cover and (2) cultivated or natural/semi-natural grassland. In order to improve the consistency of the mapping feedback and avoid ambiguities in visual interpretation and classification, users are provided with sufficient materials to follow the predefined labeling criteria and protocols. The consortium considers that systematically collected feedback, together with multiple partnerships and wide stakeholder participation, will lead to the most efficient path for improving future versions of the Global Pasture Watch products, supporting the development of fit-for-purpose applications able to advance the protection, restoration and sustainable use of global grasslands. We encourage and welcome all readers of this publication to contribute knowledge to this effort.

## Supplementary information

Supplementary information

## Data Availability

All workflow presented in this paper were implemented in Python, and the source code is publicly available (MIT License) at: https://github.com/wri/global-pasture-watch. For reproducibility purposes, we have archived a snapshot of the source code (release ggc30m_v1) (10.5281/zenodo.13952867^81^), all reference samples (10.5281/zenodo.14035457^82^) and trained models (10.5281/zenodo.13952806^83^) in Zenodo.
