# Supplementary material for: Annual 30-m maps of global grassland class and extent (2000–2022) based on spatiotemporal Machine Learning
Source: Sci Data. 2024 Dec 11;11:1303. doi: 10.1038/s41597-024-04139-6 (PMC11634896; doi:10.1038/s41597-024-04139-6)
Supplement: Supplementary file 1 — Supplementary information [file 41597_2024_4139_MOESM1_ESM.pdf]

Table S1. ESA CCI land cover classes used to establish the short vegetation mask of the Feature Space Coverage Sampling (FSCS). The mask considered all pixels mapped as one of the 13 classes in at least one years of the time series (1993 – 2021), including stable and recent converted areas of pastures / grasslands.

Code	Land cover class
30	Mosaic cropland (>50%) / natural vegetation (tree/shrub/herbaceous cover) (<50%)
40	Mosaic natural vegetation (tree/shrub/herbaceous cover) (>50%) / cropland (<50%)
100	Mosaic tree and shrub (>50%) / herbaceous cover (<50%)
110	Mosaic herbaceous cover (>50%) / tree and shrub (<50%)
120	Shrubland
121	Shrubland evergreen
122	Shrubland deciduous
130	Grassland
150	Sparse vegetation (tree/shrub/herbaceous cover) (<15%)
151	Sparse tree (<15%)
152	Sparse shrub (<15%)
153	Sparse herbaceous cover
180	Shrub or herbaceous cover flooded fresh/saline/brackish water

Table S2. Hierarchical ontology based on Allen et al., 2011 with most appropriate English terminology for Cultivated Grasslands and Natural/Semi-Natural Grasslands.

<table>
<tr><th colspan="2">Grazing land</th></tr>
<tr><td colspan="2">1.1 Grazing land (n.). Any vegetated land that is grazed or has the potential to be grazed by animals (domestic and wild). This term is all-inclusive and covers all kinds and types of land that can be grazed.</td></tr>
<tr><th colspan="2">Grassland, rangelands and pastures</th></tr>
<tr><td colspan="2">1.1.3 Grassland (n.). The term ‘grassland’ is synonymous with pastureland when referring to an imposed grazing-land ecosystem. The vegetation of grassland in this context is broadly interpreted to include grasses, legumes and other forbs, and at times woody species may be present (cf. Native or Natural Grassland, 1.1.4.2).</td></tr>
<tr><td colspan="2">1.1.4 Rangeland (n.). Land on which the indigenous vegetation (climax or sub-climax) is predominantly grasses, grass-like plants, forbs or shrubs that are grazed or have the potential to be grazed, and which is used as a natural ecosystem for the production of grazing livestock and wildlife.</td></tr>
<tr><td colspan="2">1.1.3 Pastureland (n.). Land (and the vegetation growing on it) devoted to the production of introduced or indigenous forage for harvest by grazing, cutting, or both. Usually managed to arrest successional processes (cf. Grassland, Note No. 1.1.3; Pasture, 5.3.4; Rangeland, 1.1.4).</td></tr>
<tr><th>Cultivated grasslands</th><th>Natural/Semi-Natural grasslands</th></tr>
<tr><td colspan="2">1.1.3.3 Permanent pastureland/grassland (n.). Land on which vegetation is composed of perennial or self-seeding annual forage species which may persist indefinitely. It may include either naturalized or cultivated forages.</td></tr>
<tr><td>1.1.3.2 Cultivated pastureland/grassland (n.). Forage is established with domesticated introduced or indigenous species that may receive periodic cultural treatment such as renovation, fertilization or weed control.</td><td>1.1.4.2 Native or natural grassland (n.). Natural ecosystem dominated by indigenous or naturally occurring grasses and other herbaceous species used mainly for grazing by livestock and wildlife (cf. Naturalized pastureland, 1.1.3.5; Rangeland, 1.1.4; Pastureland and Grassland, 1.1.3).</td></tr>
<tr><td>7. Stocking methods. The term ‘stocking’ is preferred to ‘grazing’ (i.e. ‘stocking method’ vs. ‘grazing method’) because grazing refers to the consumption of standing forage (cf. Graze, 3.2.1.3), whereas it is the method of stocking grazing animals that allows manipulation of how, when, what and how much the animals graze.</td><td>1.1.3.6 Semi-natural pastureland/grassland (n.). Managed ecosystem dominated by indigenous or naturally occurring grasses and other herbaceous species (cf. Native grassland, 1.1.4.2).</td></tr>
</table>

Table S3. Filtering rules applied in the point samples based on three global land cover products. The analysis considered a 5-year time series (*i.e.* 2000, 2005, 2010, 2015 and 2020) for UMD GLAD GLCLUC and GLC_FCS30D products and a single year (*i.e.* 2020) for ESA WorldCover, where all points samples were compared with two different years. For example, a sample labeled as "Cultivated grassland" in 2018 and mapped as "Cropland" by UMD GLAD GLCLUC, "Rainfed cropland" by GLC_FCS30D and "Cropland" by ESA WorldCover in 2015 and 2020 was removed. Likewise, a sample labeled as "Others" in 2003 and mapped as "Terra Firma, dense short vegetation" by UMD GLAD GLCLUC and "Grassland" by GLC_FCS30D in 2000 and 2005 was removed.

**Cultivated grassland and Natural/semi-natural grassland**		
UMD GLAD GLCLUC	GLC_FCS30D	ESA WorldCover 2020
Terra Firma, stable tree cover (25–48)<br>Cropland, Stable (244)<br>Wetland, stable tree cover (125–148)<br>Ocean (254)<br>Snow/ice, stable (241)	Forest (51,52,61,62,71,72,81,82)<br>Irrigated cropland (20)<br>Mangrove (185)<br>Permanent ice and snow (220)<br>Rainfed cropland (10)<br>Water body (210)<br>Impervious surfaces (190)<br>Consolidated bare areas (201)<br>Marsh (182)<br>Closed mixed leaf forest (92)<br>Herbaceous cover cropland (11)<br>Saline (184)<br>Salt marsh (186)<br>Swamp (181)	Tree cover (10)<br>Cropland (40)<br>Built-up (50)<br>Snow and Ice (70)<br>Permanent water bodies (80)<br>Mangroves (95)
**Other**		
UMD GLAD GLCLUC	GLC_FCS30D	ESA WorldCover 2020
Terra Firma, dense short vegetation (19–24)<br>Wetland, dense short vegetation (119–124)	Grassland (130)	Herbaceous

Table S4. List of Landsat-derived indices used by our modelling; the Bare Soil Fraction (BSF) is not included in this list given its relatively simple formulation already provided in the text.

Landsat-derived Index	Abbreviation	Formula
Bare Soil Index	BSI	$\frac{(SWIR1+RED)-(NIR+BLUE)}{(SWIR1+RED)+(NIR+BLUE)}$
Enhanced Vegetation Index	EVI	$2.5\times\frac{NIR-RED}{NIR+6\times RED-7.5\times BLUE+1}$
Fraction of Absorbed Photosynthetically Active Radiation	FAPAR	$\frac{(NDVI-0.03)\times(0.95-0.001)}{0.96-0.03}+0.001$
Normalized Difference Tillage Index	NDTI	$\frac{SWIR1-SWIR2}{SWIR1+SWIR2}$
Normalized Difference Vegetation Index	NDVI	$\frac{NIR-RED}{NIR+RED}$
Normalized Difference Water Index	NDWI	$\frac{NIR-SWIR1}{NIR+SWIR1}$
Near-infrared reflectance of vegetation	NIRv	$\left(\frac{NIR-RED}{NIR+RED}-0.8\right)\times NIR$

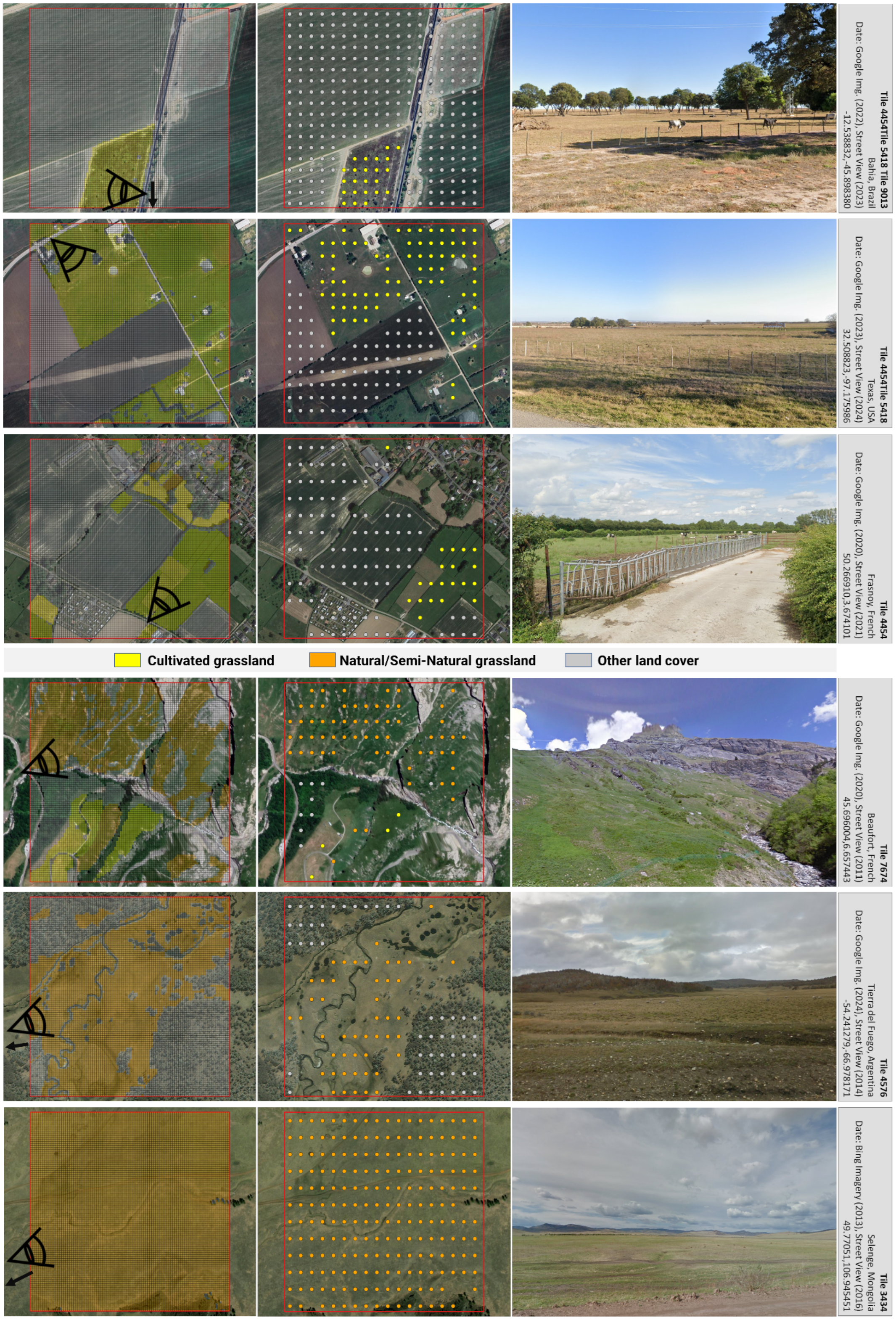

Figure S1. Visual interpretation examples for cultivated and natural/semi-natural grassland based on Google Maps, Bing Maps and Google Street View. The black line shows the view angle of the Google Street View photo and was produced by QGIS plugin Street View.

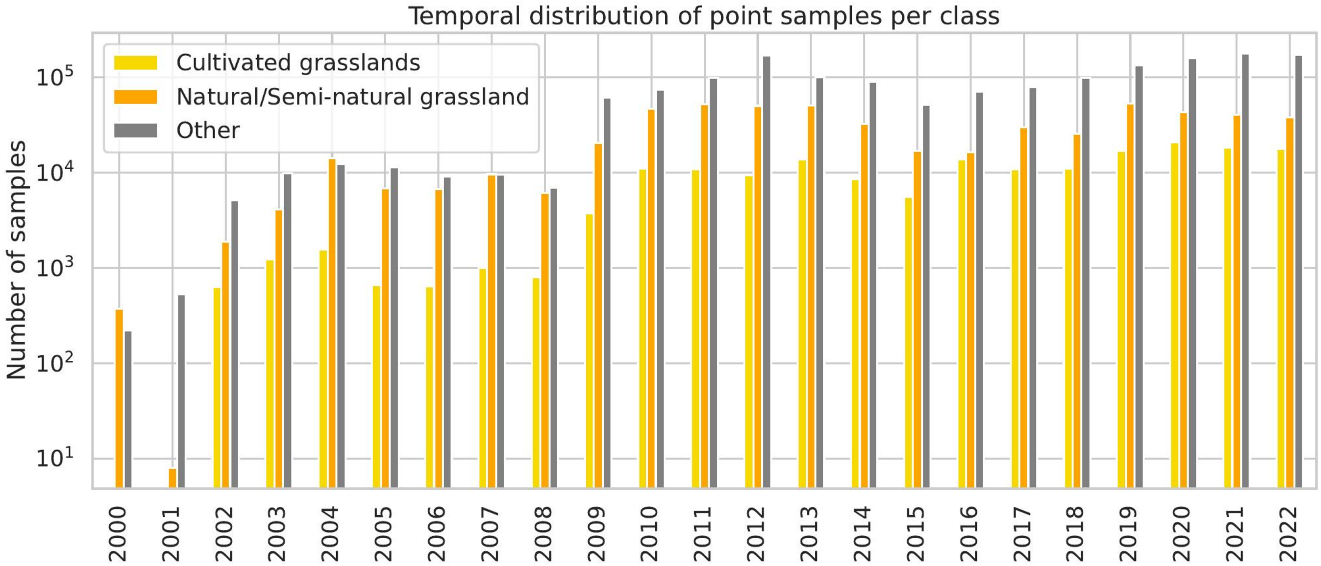

Figure S2. Temporal distribution of point samples according to the class defined by visual interpretation. Combining all the years, 68% of samples were labeled as "Others" and 24% and 8% as *Cultivated grassland* and *Natural/semi-natural grassland*, respectively.

Table S5. Feature selected by Recursive Feature Elimination - RFE, per class of grass, and used to train the final machine model. learning

#	Feature	Type	Cultivated grassland RFE	Natural / Semi-natural grass RFE
0	bsi_glad.landsat.ard2.seasconv_m_30m_s_0101_0228	Bi-monthly Landsat		X
1	bsi_glad.landsat.ard2.seasconv_m_30m_s_0301_0430	Bi-monthly Landsat		X
2	bsi_glad.landsat.ard2.seasconv_m_30m_s_0701_0831	Bi-monthly Landsat	X	
3	bsi_glad.landsat.ard2.seasconv_m_30m_s_1101_1231	Bi-monthly Landsat		X
4	lst_max.geom.temp_m_30m_s_m1	Geometric temperature	X	
5	lst_max.geom.temp_m_30m_s_m11	Geometric temperature	X	
6	lst_max.geom.temp_m_30m_s_m2	Geometric temperature	X	X
7	lst_max.geom.temp_m_30m_s_m3	Geometric temperature	X	
8	lst_max.geom.temp_m_30m_s_m9	Geometric temperature		X
9	lst_min.geom.temp_m_30m_s_m1	Geometric temperature	X	X
10	lst_min.geom.temp_m_30m_s_m5	Geometric temperature		X
11	lst_min.geom.temp_m_30m_s_m7	Geometric temperature	X	
12	lst_mod11a2.daytime.m02_p50_1km_s_20000101_20221231	Long-term MODIS	X	X
13	lst_mod11a2.daytime.m02_sd_1km_s_20000101_20221231	Long-term MODIS	X	
14	lst_mod11a2.daytime.m03_p50_1km_s_20000101_20221231	Long-term MODIS		X
15	lst_mod11a2.daytime.m03_sd_1km_s_20000101_20221231	Long-term MODIS	X	X
16	lst_mod11a2.daytime.m04_p50_1km_s_20000101_20221231	Long-term MODIS	X	X
17	lst_mod11a2.daytime.m04_sd_1km_s_20000101_20221231	Long-term MODIS	X	X
18	lst_mod11a2.daytime.m05_p50_1km_s_20000101_20221231	Long-term MODIS		X
19	lst_mod11a2.daytime.m06_p50_1km_s_20000101_20221231	Long-term MODIS	X	X
20	lst_mod11a2.daytime.m07_p50_1km_s_20000101_20221231	Long-term MODIS	X	X
21	lst_mod11a2.daytime.m07_sd_1km_s_20000101_20221231	Long-term MODIS	X	X
22	lst_mod11a2.daytime.m08_p50_1km_s_20000101_20221231	Long-term MODIS	X	X
23	lst_mod11a2.daytime.m08_sd_1km_s_20000101_20221231	Long-term MODIS	X	
24	lst_mod11a2.daytime.m09_p50_1km_s_20000101_20221231	Long-term MODIS	X	X
25	lst_mod11a2.daytime.m09_sd_1km_s_20000101_20221231	Long-term MODIS	X	
26	lst_mod11a2.daytime.m10_p50_1km_s_20000101_20221231	Long-term MODIS	X	X
27	lst_mod11a2.daytime.m10_sd_1km_s_20000101_20221231	Long-term MODIS	X	X
28	lst_mod11a2.daytime.m11_p50_1km_s_20000101_20221231	Long-term MODIS	X	X
29	lst_mod11a2.daytime.m11_sd_1km_s_20000101_20221231	Long-term MODIS	X	
30	lst_mod11a2.daytime.m12_p50_1km_s_20000101_20221231	Long-term MODIS	X	X
31	lst_mod11a2.daytime.m12_sd_1km_s_20000101_20221231	Long-term MODIS	X	
32	lst_mod11a2.nighttime.m03_p50_1km_s_20000101_20221231	Long-term MODIS	X	X
33	lst_mod11a2.nighttime.m05_p50_1km_s_20000101_20221231	Long-term MODIS	X	X
34	lst_mod11a2.nighttime.m06_p50_1km_s_20000101_20221231	Long-term MODIS		X
35	lst_mod11a2.nighttime.m07_p50_1km_s_20000101_20221231	Long-term MODIS	X	X
36	lst_mod11a2.nighttime.m07_sd_1km_s_20000101_20221231	Long-term MODIS		X
37	lst_mod11a2.nighttime.m08_p50_1km_s_20000101_20221231	Long-term MODIS	X	X

38	lst_mod11a2.nighttime.m09_p50_1km_s_20000101_20221231	Long-term MODIS	X	X
39	lst_mod11a2.nighttime.m09_sd_1km_s_20000101_20221231	Long-term MODIS	X	
40	lst_mod11a2.nighttime.m11_sd_1km_s_20000101_20221231	Long-term MODIS	X	
41	lst_mod11a2.nighttime.m12_p50_1km_s_20000101_20221231	Long-term MODIS	X	
43	dtm_elevation_merit.dem_m_250m_s0..0cm_2017	Static DTM	X	X
42	dtm.bareearth_ensemble_p10_30m_s_2018	Static DTM	X	X
44	fapar_glad.landsat.ard2.seasconv_m_30m_s_0301_0430	Bi-monthly Landsat		X
45	fapar_glad.landsat.ard2.seasconv_m_30m_s_0501_0630	Bi-monthly Landsat		X
46	fapar_glad.landsat.ard2.seasconv_m_30m_s_0701_0831	Bi-monthly Landsat		X
47	fapar_glad.landsat.ard2.seasconv_m_30m_s_1101_1231	Bi-monthly Landsat		X
48	green_glad.landsat.ard2.seasconv_m_30m_s_0501_0630	Bi-monthly Landsat	X	
49	green_glad.landsat.ard2.seasconv_m_30m_s_0701_0831	Bi-monthly Landsat	X	
52	lcv_accessibility.to.cities_map.ox.var1_m_1km_s0..0cm_2015	Static distance maps	X	X
50	lcv_accessibility.to.cities_map.ox.var11_m_1km_s0..0cm_2015	Static distance maps	X	X
51	lcv_accessibility.to.cities_map.ox.var12_m_1km_s0..0cm_2015	Static distance maps		X
53	lcv_accessibility.to.cities_map.ox.var2_m_1km_s0..0cm_2015	Static distance maps	X	X
54	lcv_accessibility.to.cities_map.ox.var3_m_1km_s0..0cm_2015	Static distance maps	X	X
55	lcv_accessibility.to.cities_map.ox.var4_m_1km_s0..0cm_2015	Static distance maps	X	X
56	lcv_accessibility.to.cities_map.ox.var5_m_1km_s0..0cm_2015	Static distance maps	X	X
57	lcv_accessibility.to.cities_map.ox.var6_m_1km_s0..0cm_2015	Static distance maps		X
58	lcv_accessibility.to.cities_map.ox.var7_m_1km_s0..0cm_2015	Static distance maps		X
59	lcv_accessibility.to.cities_map.ox.var8_m_1km_s0..0cm_2015	Static distance maps	X	X
60	lcv_accessibility.to.cities_map.ox.var9_m_1km_s0..0cm_2015	Static distance maps	X	X
61	lcv_road.distance_osm.highways.high.density_pxl_100m_0..0cm_2022	Static distance maps		X
62	ndti_glad.landsat.ard2.seasconv_m_30m_s_0301_0430	Bi-monthly Landsat	X	
63	ndti_glad.landsat.ard2.seasconv_m_30m_s_0501_0630	Bi-monthly Landsat	X	
64	ndti_glad.landsat.ard2.seasconv_m_30m_s_0701_0831	Bi-monthly Landsat	X	
65	ndti_glad.landsat.ard2.seasconv_m_30m_s_0901_1031	Bi-monthly Landsat	X	
66	ndti_glad.landsat.ard2.seasconv_m_30m_s_1101_1231	Bi-monthly Landsat	X	
67	ndvi_glad.landsat.ard2.seasconv_m_30m_s_0101_0228	Bi-monthly Landsat		X
68	ndvi_glad.landsat.ard2.seasconv_m_30m_s_0701_0831	Bi-monthly Landsat		X
69	ndvi_glad.landsat.ard2.seasconv_m_30m_s_1101_1231	Bi-monthly Landsat		X
70	ndwi_glad.landsat.ard2.seasconv_m_30m_s_0101_0228	Bi-monthly Landsat		X
71	ndwi_glad.landsat.ard2.seasconv_m_30m_s_0501_0630	Bi-monthly Landsat		X
72	ndwi_glad.landsat.ard2.seasconv_m_30m_s_0701_0831	Bi-monthly Landsat	X	X
73	ndwi_glad.landsat.ard2.seasconv_m_30m_s_1101_1231	Bi-monthly Landsat		X
74	red_glad.landsat.ard2.seasconv_m_30m_s_0501_0630	Bi-monthly Landsat		X
75	red_glad.landsat.ard2.seasconv_m_30m_s_0701_0831	Bi-monthly Landsat	X	X
76	red_glad.landsat.ard2.seasconv_m_30m_s_0901_1031	Bi-monthly Landsat		X
77	red_glad.landsat.ard2.seasconv_m_30m_s_1101_1231	Bi-monthly Landsat		X
78	swir1_glad.landsat.ard2.seasconv_m_30m_s_0101_0228	Bi-monthly Landsat	X	
79	swir1_glad.landsat.ard2.seasconv_m_30m_s_0301_0430	Bi-monthly Landsat	X	

80	swir1_glad.landsat.ard2.seasconv_m_30m_s_0501_0630	Bi-monthly Landsat	X	X
81	swir1_glad.landsat.ard2.seasconv_m_30m_s_0701_0831	Bi-monthly Landsat	X	X
82	swir1_glad.landsat.ard2.seasconv_m_30m_s_0901_1031	Bi-monthly Landsat	X	
83	swir1_glad.landsat.ard2.seasconv_m_30m_s_1101_1231	Bi-monthly Landsat	X	X
84	swir2_glad.landsat.ard2.seasconv_m_30m_s_0501_0630	Bi-monthly Landsat	X	
85	wv_mcd19a2v061.seasconv.m.m01_p50_1km_s_20000101_20221231	Long-term MODIS	X	X
86	wv_mcd19a2v061.seasconv.m.m02_p50_1km_s_20000101_20221231	Long-term MODIS	X	X
87	wv_mcd19a2v061.seasconv.m.m03_p50_1km_s_20000101_20221231	Long-term MODIS	X	
88	wv_mcd19a2v061.seasconv.m.m04_sd_1km_s_20000101_20221231	Long-term MODIS	X	
89	wv_mcd19a2v061.seasconv.m.m05_p50_1km_s_20000101_20221231	Long-term MODIS		X
90	wv_mcd19a2v061.seasconv.m.m05_sd_1km_s_20000101_20221231	Long-term MODIS	X	X
91	wv_mcd19a2v061.seasconv.m.m06_p50_1km_s_20000101_20221231	Long-term MODIS	X	X
92	wv_mcd19a2v061.seasconv.m.m07_p50_1km_s_20000101_20221231	Long-term MODIS	X	X
93	wv_mcd19a2v061.seasconv.m.m07_sd_1km_s_20000101_20221231	Long-term MODIS	X	
94	wv_mcd19a2v061.seasconv.m.m08_p50_1km_s_20000101_20221231	Long-term MODIS	X	X
95	wv_mcd19a2v061.seasconv.m.m08_sd_1km_s_20000101_20221231	Long-term MODIS	X	X
96	wv_mcd19a2v061.seasconv.m.m09_p50_1km_s_20000101_20221231	Long-term MODIS		X
97	wv_mcd19a2v061.seasconv.m.m09_sd_1km_s_20000101_20221231	Long-term MODIS	X	X
98	wv_mcd19a2v061.seasconv.m.m10_p50_1km_s_20000101_20221231	Long-term MODIS	X	X
99	wv_mcd19a2v061.seasconv.m.m10_sd_1km_s_20000101_20221231	Long-term MODIS	X	X
100	wv_mcd19a2v061.seasconv.m.m11_p50_1km_s_20000101_20221231	Long-term MODIS	X	X
101	wv_mcd19a2v061.seasconv.m.m12_p50_1km_s_20000101_20221231	Long-term MODIS	X	X
102	wv_mcd19a2v061.seasconv.m.m12_sd_1km_s_20000101_20221231	Long-term MODIS	X	

Table S6. Final hyper-parameters selected by Successive Halving based on the best `log_loss` and a five-fold spatial cross-validation by sample tile (*i.e.* 1 x 1 km) using the calibration set.

Cultivated grassland			
Algorithm	**Random Forest**	**Gradient-boosted trees**	**Artificial neural network**
Implementation	RandomForestClassifier (Scikit-learn)	XGBClassifier (XGBoost)	MLPClassifier (Scikit-learn)
Best log_loss	0.177	0.172	0.183
Hyper-params.	class_weight=None<br>criterion=entropy<br>max_depth=56<br>max_features=0.1807<br>min_samples_leaf=7<br>min_samples_split=37	alpha=1.052<br>colsample_bylevel=0.670<br>colsample_bynode=0.201<br>colsample_bytree=0.958<br>eta=1.435<br>gamma=1.944<br>grow_policy=depthwise<br>learning_rate=0.089<br>max_depth=72<br>n_estimators=57<br>reg_alpha=0.152<br>reg_lambda=0.013<br>subsample=0.993<br>tree_method=hist	activation=logistic<br>alpha=0.0001<br>batch_size=53<br>beta_1=0.898<br>beta_2=0.716<br>epsilon=1.054e-08<br>hidden_layer_sizes=(7,224)<br>learning_rate=adaptive<br>learning_rate_init=0.0010<br>solver=adam
**Natural/semi-natural grassland**			
Algorithm	**Random Forest**	**Gradient-boosted trees**	**Artificial neural network**
Implementation	RandomForestClassifier (Scikit-learn)	XGBClassifier (XGBoost)	MLPClassifier (Scikit-learn)
Best log_loss	0.295	0.289	0.310
Hyper-params.	class_weight=None<br>criterion=entropy<br>max_depth=96<br>max_features=0.221<br>min_samples_leaf=6<br>min_samples_split=22	alpha=0.046<br>colsample_bylevel=0.533<br>colsample_bynode=0.643<br>colsample_bytree=0.600<br>eta=1.058<br>gamma=1.992<br>grow_policy=lossguide<br>learning_rate=0.080<br>max_depth=51<br>n_estimators=56<br>reg_alpha=0.0267<br>reg_lambda=0.192<br>subsample=0.673<br>tree_method=approx	activation=relu<br>alpha=0.0001<br>batch_size=179<br>beta_1=0.762<br>beta_2=0.939<br>epsilon=1.096e-08<br>hidden_layer_sizes=(7,128)<br>learning_rate=constant<br>learning_rate_init=0.0008<br>solver=adam

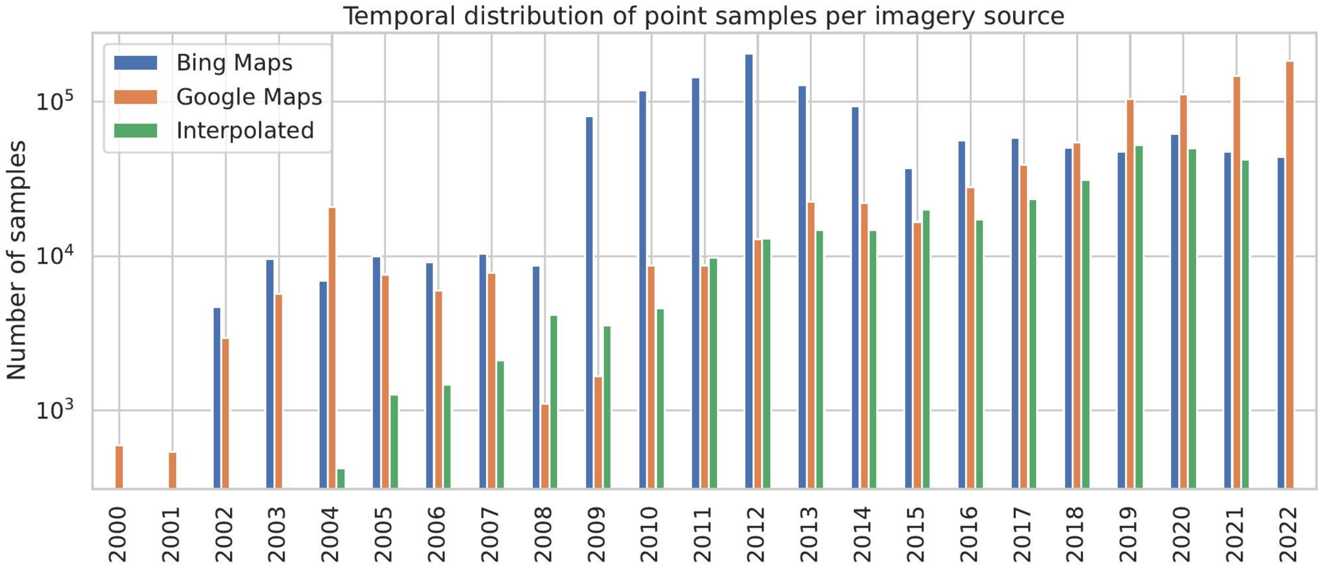

Figure S3. Temporal distribution of point samples per imagery source used in the visual interpretation process. Combining all the years, 52% of samples were based on Bing Maps, 35% in Google Maps and 13% were interpolated by our preprocessing approach.

Table S7. Land cover classes mapped by UMD GLAD GLCLUC product and used to establish our prediction mask. All pixels mapped as one of the listed classes in all years between 2000–2020 were ignored by our global predictions.

Codes	Land cover class
0–1	Terra firme, true desert
2–5	Semi-arid with less than 24% of vegetation cover
32–48	Stable tree cover with trees higher than 9 m
100–101	Wetland, salt pan with less than 8% of vegetation cover
241	Stable snow/ice
254	Ocean water

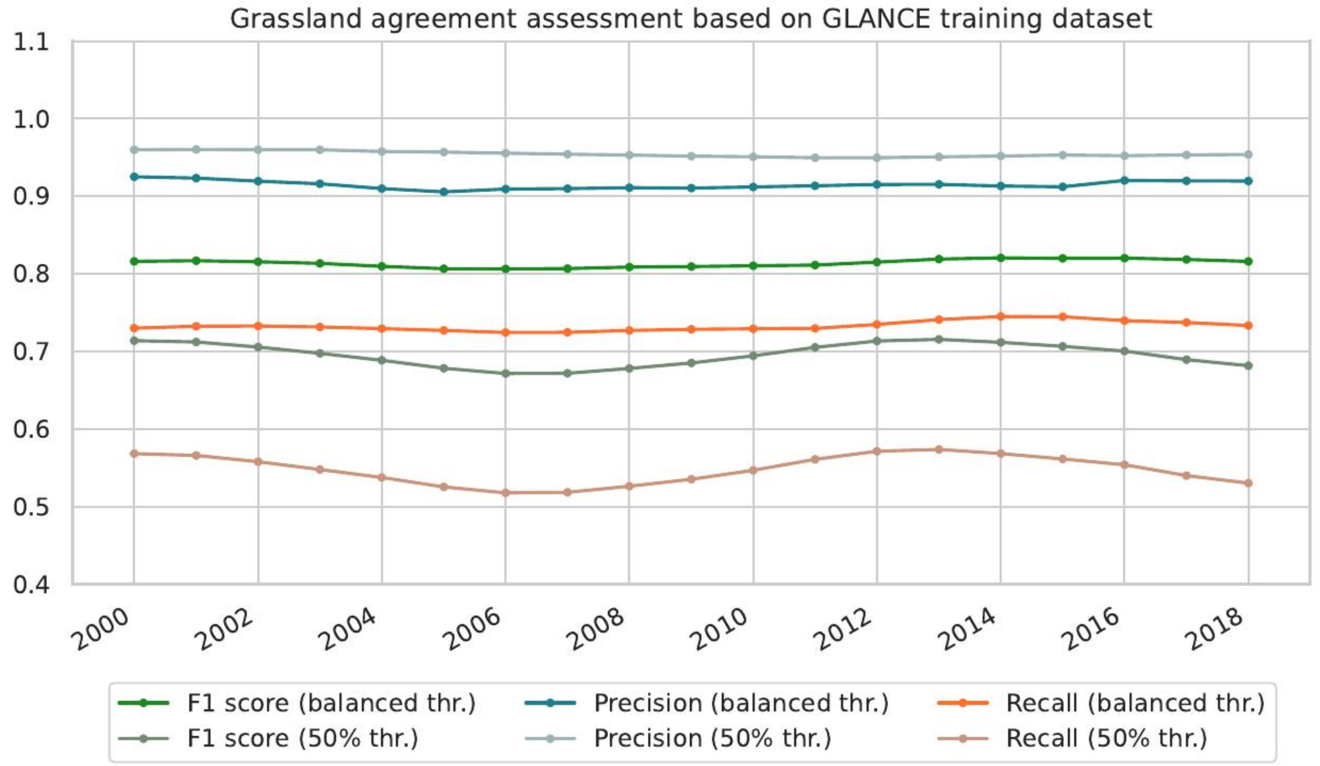

Figure S4. Accuracy assessment of grassland class (*i.e.* cultivated and natural / semi-natural grassland combined) based on GLANCE training dataset. The GLANCE classes grassland (12), shrub (10) and moss / lichen (13) were reclassified to grassland for matching with our legend. All metrics were derived considering naive threshold (*i.e.* 0.5 for both classes) and balanced thresholds of 0.32 and 0.42 for cultivated and natural / semi-natural grassland, respectively.
